# CottonFGD: an integrated functional genomics database for cotton

**DOI:** 10.1186/s12870-017-1039-x

**Published:** 2017-06-08

**Authors:** Tao Zhu, Chengzhen Liang, Zhigang Meng, Guoqing Sun, Zhaoghong Meng, Sandui Guo, Rui Zhang

**Affiliations:** Biotechnology Research Institute, Chinese Academy of Agricultural Sciences, Beijing, 100081 China

**Keywords:** Cotton, Database, RNA-seq, Functional annotation, Variation, Genetic marker

## Abstract

**Background:**

Cotton (*Gossypium* spp.) is the most important fiber and oil crop in the world. With the emergence of huge -omics data sets, it is essential to have an integrated functional genomics database that allows worldwide users to quickly and easily fetch and visualize genomic information. Currently available cotton-related databases have some weakness in integrating multiple kinds of -omics data from multiple *Gossypium* species. Therefore, it is necessary to establish an integrated functional genomics database for cotton.

**Description:**

We developed CottonFGD (Cotton Functional Genomic Database, https://cottonfgd.org), an integrated database that includes genomic sequences, gene structural and functional annotations, genetic marker data, transcriptome data, and population genome resequencing data for all four of the sequenced *Gossypium* species. It consists of three interconnected modules: search, profile, and analysis. These modules make CottonFGD enable both single gene review and batch analysis with multiple kinds of -omics data and multiple species. CottonFGD also includes additional pages for data statistics, bulk data download, and a detailed user manual.

**Conclusion:**

Equipped with specialized functional modules and modernized visualization tools, and populated with multiple kinds of -omics data, CottonFGD provides a quick and easy-to-use data analysis platform for cotton researchers worldwide.

**Electronic supplementary material:**

The online version of this article (doi:10.1186/s12870-017-1039-x) contains supplementary material, which is available to authorized users.

## Background

As a natural fiber and oilseed crop, cotton (*Gossypium* spp.) plays an important role in daily life and industrial material. In addition, the polyploidy of currently cultivated cottons, and its close relationship with ancestral diploid donor species makes it an excellent model organism for studies of polyploidization. These two aspects have resulted in demand for an integrated genomics database that provides gene information resources for researchers engaged in molecular breeding and in evolutionary studies.

Compared with other model organisms such as *Arabidopsis thaliana*, rice (*Oryza sativa*), and maize (*Zea mays*), the genome sequences of cotton species were released much later. The first cotton genome assembly for *G. raimondii*, a diploid species that donated the D-subgenome of cultivated polyploid cotton, was released in 2012 by two independent groups [1, 2]. Genomes of three other important cotton species, *G. arboreum* (diploid), *G. hirsutum* and *G. barbadense* (both polyploid), were just released in the last two years [3–7] (See review [8] for details). Likely due to this rather late start, the information about cotton genomics is not readily available in popular general plant sequence databases. Among the 58 general plant databases included in the *Nucleic Acids Research* Molecular Biology Database Collection [9], only seven include cotton genes’ information. Moreover, among these, six only include data for a single diploid species, *G. raimondii*..

In addition to the general plant databases, there are also three databases specifically designed for cotton. CottonGen [10] collects cotton genome sequences, genetic markers, and breeding germplasm accessions. GraP [11] is a *G. raimondii-*specific database for gene functional annotation and expression data. ccNet [12] displays co-expression networks from diploid *G. arboreum* and polyploid *G. hirsutum*. While these databases filled in many gaps in cotton genome and -omics data analysis, the decentralized distribution of these databases make it a complex task to access this information in the course of practical research work. Researchers need ready access to a variety data types from multiple *Gossypium* species, including information relating to genetics, genomics, functional annotations, transcriptomics and sequence variation data. Thus, an integrated functional genomics database similar to the IC4R rice database [13] is necessary to systematically gather current cotton genomics data together for easy use.

Here, we developed CottonFGD, an integrated functional genomics database for cotton. CottonFGD features three notable attributes: comprehensiveness, integrity, and user-friendliness. First, it covers all of the available cotton genomes and a variety of genetics and -omics data, including genetic marker annotations, structural annotations, functional annotations, RNA-seq expression data sets, and population resequencing data. Second, CottonFGD integrates gene searching, cross-database referencing, and gene list analysis in an easy and natural way. Last, but not least, CottonFGD employs modern visualization tools that make its user interface accessible via any type of device. We hope that CottonFGD will emerge as the fundamental database for the cotton functional genomics and breeding research community.

## Construction and content

### Data sources and processing

#### Genome assemblies and gene annotations

Seven cotton genome assemblies representing four *Gossypium* species and their respective gene annotations were downloaded from relevant database websites (Additional file 1). After checking the annotation consistency between the GFF files and the provided CDS or protein sequences, we found that the HAU assembly (v1.0) and annotation (v1.0) of *G. barbadense* [6] contain systemic errors; it was therefore not included in CottonFGD (Additional file 1). In total, six assemblies were used in CottonFGD (Table 1). In order to make the annotation data from different species more consistent, several subtle changes were implemented (Additional file 1). All the patched annotation files are available for download from CottonFGD.

**Table 1:** Cotton genome assemblies included in CottonFGD

Species^a^	Date Provider	Assembly Size (Mb)	Chromosome Number^b^	Annotated Genes
Diploid				
*G. raimondii* (Ulbr.)	Joint Genome Institute (JGI) [1]	761.4	13 (+1020)	37,505
*G. raimondii* (D_5_–3)	Beijing Genome Institute (BGI) [2]	775.2	13 (+4434)	40,976
*G. arboreum* (Shixiya1)	Beijing Genome Institute (BGI) [3]	1694.6	13 (+75,581)	41,331
Tetraploid				
*G. hirsutum* (Tm-1)	Nanjing Agricultural University (NAU) [7]	2447.0	26 (+38,951)	70,478
*G. hirsutum* (Tm-1)	Beijing Genome Institute (BGI) [4]	2150.9	26 (+9128)	76,943
*G. barbadense* (Xinhai-21)	Nanjing Agricultural University (NAU) [5]	2263.5	26 (+2013)	77,358

^a^Sequenced strains are listed in brackets.

^b^Unplaced scaffold numbers are listed in brackets

#### Gene functional annotations

Each gene name and description was defined by its best protein homolog from NCBI BLAST+ [14] (v2.2.31) searching against the UniProtKB/SwissProt database [15] (last accessed December, 2015) with an e-value of 1e-05. Predicted protein properties such as molecular weight, isoelectric point, and hydropathy were calculated using EMBOSS [16] (v6.5.7.0) and BioPerl [17] (v1.6.924). Included protein motif/domain regions and associated Gene Ontology [18] (GO) and InterPro [19] items were annotated using InterProScan [20] (v5.16–55.0) with the default parameters. Related pathways were annotated using the KEGG Automatic Annotation Server [21] (KAAS) with the bi-directional best hit method, against of all the available plant species. Homologs within *Gossypium* and across other representative plant species were defined by BLAST+ with e-values of 1e-10 and 1e-5, respectively. In addition, we also collect functional annotation data from the original sequencing projects and the CottonGen [10] database. Detailed data source can be viewed from the help document for CottonFGD (https://cottonfgd.org/about/help/).

#### Genetic Marker Annotations

Genetic marker sequences of 279 insertion/deletion sites (INDELs), 3451 restricted fragment length polymorphisms (RFLPs), and 65,412 simple sequence repeats (SSRs) were downloaded from CottonGen [10]. Each marker was mapped to every *Gossypium* genome assembly to define its physical location using BLAT [22] (v36). By default, only BLAT hits with ≥95% query coverage and ≥90% identity were shown in the final user interface.

#### Expression data

By searching the Sequence Read Archive [23] (SRA) database of NCBI, we collected and downloaded 168 RNA-seq analyses, the majority of which had more than 20× transcriptome sequencing depth and read lengths longer than 75 bp. These RNA-seq analyses constitute 20 experiment groups (Additional file 2) covering all four of the *Gossypium* species in CottonFGD, and cover a variety of biological processes like stress responses and developmental series such as seed germination and fiber development, as well as multiple tissue expression atlases. Raw RNA-seq reads were filtered using the NGS QC Toolkit [24] (v2.3.3) and were then trimmed by Trimmomatic [25] (v0.3.3) to generate clean reads for further analysis. The resulting clean RNA-seq reads were mapped to their respective reference genomes using TopHat [26] (v2.1.1). The transcript abundance of annotated genes was quantified by Cufflinks [27] (v2.2.1) and then the differentially-expressed genes (DEGs) were defined within each experiment group. Detailed parameters for the software used here are listed in the help document for CottonFGD (https://cottonfgd.org/about/help/).

#### Variation data

Whole Genome Shot-gun (WGS) resequencing data were also searched and downloaded from the NCBI SRA database. 122 WGS analyses containing 85 *G. hirsutum* strains and 103 analyses containing 57 *G. barbadense* strains were selected (both datasets were from study SRP047301). Raw WGS reads were filtered using the same methods used for our filtering of RNA-seq reads. The filtered reads were mapped to the relevant reference genomes using BWA [28] (v0.7.12). In order to reduce false positive variant calling, we only used WGS analyses with more than 50% clean reads remaining after quality filtering and for which more than 80% of reads were properly mapped. These criteria yielded 96 analyses containing 79 *G. hirsutum* strains and 83 analyses containing 52 *G. barbadense* strains (Additional file 3). SNPs and INDELs were called using Samtools [29] (v1.3) and Bcftools [29] (v1.3). The possible effects of SNPs were annotated using SnpEff [30] (v4.3). Detailed parameters for this analysis pipeline are listed in the help document for CottonFGD (https://cottonfgd.org/about/help/).

### Development of database and webserver

The processed sequence, annotation, expression, and variation data were stored in our MySQL (v5.6.26) server. A user-friendly web interface was constructed to enable end users to conveniently access CottonFGD data. The web interface was developed using the Twitter Bootstrap framework based on modern HTML5 and JavaScript. This enables users to access CottonFGD through any modern browser on any kind of device. Multiple JavaScript tools were used to visualize the searched data (See the Utility and discussion section for details). PHP (v5.6.6) was used to submit users’ query searches and to dynamically generate report pages. Both the database and the website are hosted on our Supermicro® server running CentOS 6.8.

### Website structure

**Fig. 1:**
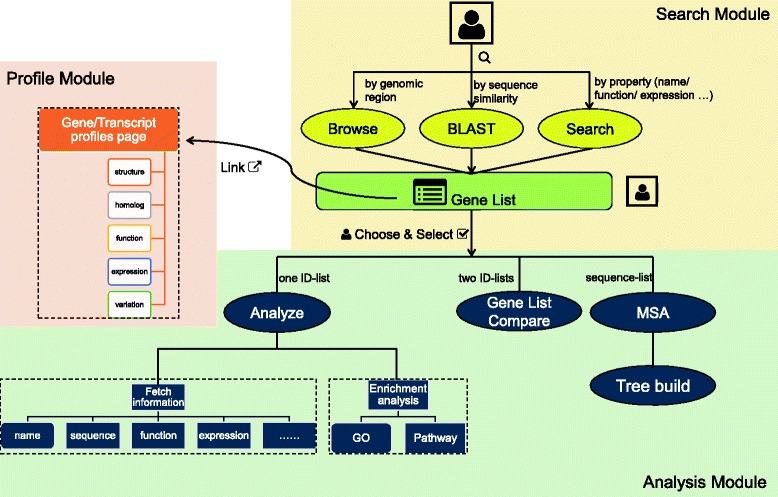
The website structure of CottonFGD. CottonFGD consists of three main modules: search, profile, and analysis. The search module accepts users’ queries and searches for cotton genes by genomic region, sequence similarity, or gene properties. The profile module displays an information page for a specified gene or transcript, including multiple properties such as gene structure, homology, gene function, and expression and sequence variation data. The analysis module can accept a list of gene IDs and generate relevant information lists; it can also conduct analyses of entire gene sets

The main structure of CottonFGD is shown in Fig. 1. It consists of three main modules: search, profile, and analysis. The search module gives users three methods to search for cotton genes: browsing by genomic regions (the “Browse” page), searching by sequence similarity (the “BLAST” page), and searching by gene properties such as names, associated domains, or expression patterns (the “Search” page). After receiving users’ queries, the search module generates a list of cotton genes as results. Users can then either click the attached link in each gene to view the relevant profile page one-by-one, or they can choose and select multiple gene IDs from the lists and launch the analysis module. In the analysis module, users can fetch information for every selected gene or conduct analysis of selected gene sets. Such analysis includes enrichment analysis, multiple sequence alignment (MSA) & phylogenetic tree construction, or gene lists comparison. All three of the modules are integrated by hyperlinks and action buttons. Therefore, it is also feasible to use CottonFGD on hand-held devices such as mobile phones, where it is not as easy to do copy and paste as it is on personal computers.

## Utility and discussion

### The search module: browse, BLAST, or search cotton genes

CottonFGD provides three methods to search for cotton genes: by genomic regions, by sequence similarity, or by gene properties.

**Fig. 2:**
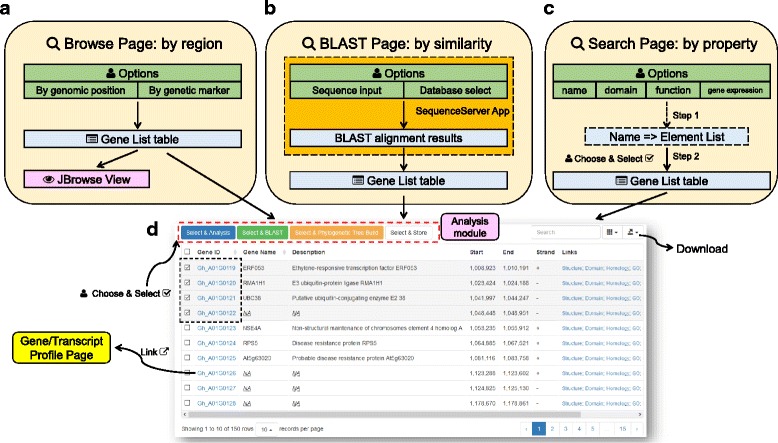
Structure of the search module. **a** The Browse page: search by genomic region (position or marker); (**b**) The BLAST page: search by sequence similarity through an embedded SequenceServer App [32]. **c** The Search page: search by names, function, or expression; (**d**) A snapshot of an interactive result table. Users can either click the hyperlink in each gene ID to view the relevant profile page or can choose and select multiple gene IDs to import into the analysis module

The “Browse page” (Fig. 2a and Additional file 4) displays annotated cotton genes in a specified genomic region. When first visiting the Browse page, it automatically displays all the annotated genes located from A01: 1,000,000–3,000,000 of the NAU assembly for *G. hirsutum*). Users can change the target species and the genomic regions to whatever they want, and can update the displayed gene lists. Regions can be defined by either genomic coordinates (physical position) or genetic markers (map position). User-altered parameters are stored in the users’ web browsers, and are automatically applied at the time of the next visit. In addition to the gene list table, CottonFGD also displays a snapshot of the gene distribution pattern in the current specified region rendered by JBrowse [31], a modern genome browser.

The “BLAST page” (Fig. 2b and Additional file 4) conducts sequence similarity searches against cotton gene sets or whole genome sequences. CottonFGD uses the latest stable version of NCBI BLAST+ [14] (currently v2.5.0) as the backend BLAST executable program and the SequenceServer app [32] (v1.0.8) as the frontend interface. This makes BLAST searching fast, stable, and appealing.

The “Search page” (Fig. 2c and Additional file 4) conducts gene searches using a variety of methods, including: by gene ID or name, by associated domains, by gene function items (GO, InterPro, or pathway), or by selected expression experiments. Users can switch among different search methods using the navigation tabs. When searching by domains or gene function names, CottonFGD implements a two-step search (Fig. 2c and Additional file 4): in the first step, CottonFGD lists all the function items that matched a user’s input. In the second step, users select the sub-items they want, and CottonFGD then returns a final associated gene list. This type of two-step searching method greatly reduces the number of redundant results that can arise from fuzzy matching of users’ search terms.

In all three of the search methods, CottonFGD renders search results in an interactive gene list table (Fig. 2d). Users can view each gene or transcript profile by clicking the relevant hyperlink in the gene ID, can download the table to their local devices in one of several formats, or can select the genes they want and do further analysis by clicking on relevant buttons located above the result table.

### The profile module: view gene/transcript profiles

Each annotated gene and its main transcript has a profile page in CottonFGD where a variety of related information is displayed. It can be accessed by hyperlinks in the search result tables or directly by input URLs. For example, the profile page of gene Gh_A01G0139 in *G. hirsutum* can be accessed via https://cottonfgd.org/profiles/gene/Gh_A01G0139/, and its main transcript Gh_A01G0139.1 can be accessed via https://cottonfgd.org/profiles/transcript/Gh_A01G0139.1/.

**Fig. 3:**
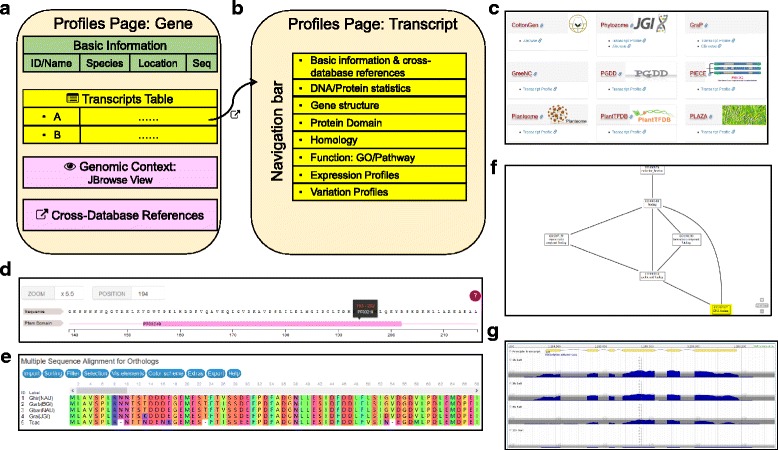
Structure of the profile module. **a** Structure of the gene profile page. Associated transcripts can be viewed in embedded tables. **b** Structure of the transcript profile page, including a variety of functional and -omics data. **c** A snapshot of cross-database references for transcripts in *G. raimondii* (See Table 2 for full cross-database reference lists). **d** A snapshot showing the Myb-like DNA-binding domain (PF00249) region of the predicted *G. hirsutum* protein Gh_A01G0139.1 (*GLK1*), rendered by the BioJS [33] feature-viewer plugin. **e** A snapshot showing the alignment of *GLK1* orthologs in four *Gossypium* species and the outgroup *Theobroma cacao*, rendered by the MSAViewer plugin [34]. **f** A snapshot showing the relationship between GO item GO:0003677 and its parent elements, rendered by the AmiGO service [35]. **g** A snapshot showing RNA-seq read coverage for *G. hirsutum* transcript Gh_A01G0139.1 (*GLK1*) in samples harvested following 1 h, 3 h, 6 h, and 12 h under salt-treated conditions, rendered by JBrowse

The profile page for a given gene displays basic information (name, description, location, and genomic DNA sequence), associated transcripts, genomic context, and cross-database references (Fig. 3a and Additional file 5). Currently, only genes from *G. raimondii* have annotation for multiple predicted isoforms; the default for this species in CottonFGD is to select the longest isoform as the principle transcript. The genomic context row displays nearby genes in surrounding 10 kb genome regions that are rendered as snapshots by JBrowse. The cross-database reference row provides relevant links to the three other cotton-specific databases and to seven general plant databases (Table 2, Fig. 3c, and Additional file 5).

**Table 2:** Cross-database references in CottonFGD

Database Name	Description	Available Genome Assemblies
Cotton specific databases		
CottonGen [10]	A genomics, genetics and breeding database for cotton research	*G. raimondii* (JGI & BGI)
		*G. arboreum* (BGI)
		*G. hirsutum* (NAU & BGI)
GraP [11]	Platform of Functional Genomics Analysis in *Gossypium raimondii*	*G. raimondii* (JGI)
ccNet [12]	Database of co-expression networks for diploid and polyploid *Gossypium*	*G. arboreum* (BGI)
		*G. hirsutum* (NAU)
General plant databases		
Phytozome [41]	A comparative platform for green plant genomics	*G. raimondii* (JGI)
GreeNC [42]	A Wiki-database of plant lncRNAs	*G. raimondii* (JGI)
PGDD [43]	Plant Genome Duplication Database	*G. raimondii* (JGI)
PIECE [44]	Plant gene structure comparison and evolution database	*G. raimondii* (JGI)
Planteome [45]	Plant Ontology database	*G. raimondii* (JGI)
PlantTFDB [46]	Plant Transcription Factor Database	*G. raimondii* (JGI)
		*G. arboreum* (BGI)
		*G. hirsutum* (NAU)
PLAZA [47]	An access point for plant comparative genomics	*G. raimondii* (JGI)

The transcript profile page displays a batch of information related to its structure, homology, function, expression, and sequence variation (polymorphisms), each in a single sub-page that can be switched via navigation tabs (Fig. 3b and Additional file 5). CottonFGD employs multiple JavaScript plugins and our own PHP scripts to visualize data. The domain regions in the protein sequence are rendered by the BioJS [33] feature-viewer plugin (Fig. 3d and Additional file 5). The multiple sequence alignment of corresponding orthologous proteins can be displayed interactively via the MSAViewer plugin [34] (Fig. 3e and Additional file 5). The network relationships among associated GO items are shown with the AmiGO service [35] (Fig. 3f and Additional file 5). The RNA-seq coverage reflecting expression levels among different samples are snapshotted by JBrowse (Fig. 3g and Additional file 5).

### The analysis module: fetch information lists or conduct set analysis

Besides viewing gene/transcript profiles one-by-one, users can also input sets of gene/transcript IDs to the analysis module and fetch their information or can conduct further analysis on a whole gene set. The query IDs can be produced either from the aforementioned search module or directly from users’ input. CottonFGD provides three methods to analyze cotton genes: by a set of gene/transcript IDs, by two sets of IDs, and by multiple sequences.

**Fig. 4:**
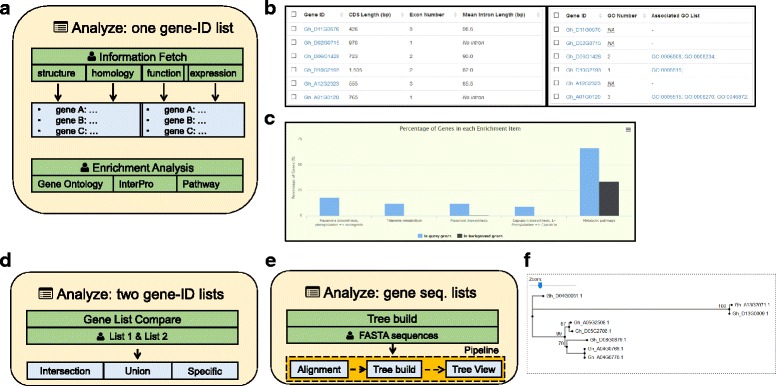
Structure of the analysis module. **a** Structure of the Analyze page: it accepts a set of gene IDs as input and fetches information or performs enrichment analysis; **(b)** A snapshot of information fetching (transcript structures and GO annotation) for six *G. hirsutum* genes. Different result types are given in the same order and can thus be easily connected from separate analyses by end users. **c** A snapshot of pathway enrichment analysis for 96 *G. hirsutum* genes using *P* < 0.0001 as a threshold, resulting in five enriched KEGG pathways. **d** Structure of the Gene List Compare page. **e** Structure of the tree build page. **f** Snapshot of an example phylogenetic tree built for six *BZIP60* and two *BZIP17* genes in *G. hirsutum*. The supporting values of tree nodes are shown in percentages

The “Analyze page” (Fig. 4a and Additional file 6) accepts a set of gene/transcript IDs as input and fetches a variety of information about gene structure, homology, function, or expression. All fetched results are grouped in a table in the same order as the user’s input. Therefore, users can easily connect results from different categories together (Fig. 4b and Additional file 6). In addition to fetching information tables, users can also do GO/InterPro/pathway enrichment analysis on specified genes (Fig. 4c and Additional file 6). Function items enriched in query genes are listed as output, and these lists are ordered by FDR corrected *P*-values calculated from the hypergeometric distribution. An interactive column chart representing the proportion of each item in the query and background genes are drawn by the HighCharts [36] tool (v4.2.0).

The “Gene List Compare page” (Fig. 4d and Additional file 6) provides a smart tool to compare two gene lists and generate their intersections, unions, or specific elements. Query IDs can be inputted directly or as stored IDs from the search module. This tool makes it easy to generate genes under complex search conditions.

The “Tree build page” (Fig. 4e and Additional file 6) contains a simple phylogenetic tree construction tool. It accepts multiple sequences in FASTA format. They are aligned using MAFFT [37] (v7.305), and the aligned sequences are clustered by FastTree [38] (v2.1.9), which is a fast and accurate tool for inferring maximum-likelihood (ML) phylogenetic trees. The output Newick tree is then visualized by the Phylo.io [39] tool (Fig. 4f and Additional file 6). Both the MSA result and the tree file can be downloaded for further use.

### Bulk data download, statistics information and user manual

Beyond the three main interactive modules, CottonFGD also includes several pages for downloading data, displaying statistical information, and database help documents. In the data download page, users can download processed data (genome assemblies, gene and protein sequences, gene annotations, expression levels, merged transcripts from RNA-seq data, etc.) in FASTA, GFF, or tab-delimited table formats. All data files are compressed to accelerate downloading, and are validated by their attached MD5 values. The statistics page present general statistics data on genome assemblies, gene models, homology, expression, and sequence variation in each species in data tables and/or interactive charts. Detailed user manuals containing data resources, data processing methods/commands, snapshots, and usage documents are also provided in CottonFGD and are linked to relevant pages.

### Limitations and future development

Due to the limitations of current assemblies and annotations, there is still some functional genomics information that not comprehensively available for all of the species included in CottonFGD. For example, alternative spliced isoforms and non-coding RNA genes are not annotated in most cotton species. In addition, the draft assemblies with large numbers of unplaced scaffolds make it difficult to precisely analyze NGS reads, leading to some inevitable artefacts when producing expression or sequence variation data. Future development of CottonFGD will proceed in two directions. On the one hand, the usage of single molecule sequencing (PacBio sequencing) and optical mapping (BioNano sequencing) will help resolve the complicated allopolyploidy of these genomes and promise to greatly improve the quality of the current assemblies. Thus, all of the current structural and functional annotations, as well as the expression and sequence variation data, will almost certainly be improved in the future. Similar sequencing methods have already been used in the allopolyploid *Brassica juncea* [40]. On the other hand, novel functional genomics data such as information about non-coding RNA gene annotations, DNA-methylation, protein interaction, etc., will be included in future iterations of CottonFGD based on the newly released public data and data from studies from our research group.

## Conclusions

CottonFGD integrates genome sequences, gene structural and functional annotations, genetic marker data, and high throughput transcriptome and WGS resequencing data in a visualized and interactive way. It provides powerful search and analysis tools to let users find and analyze their target genes easily. We anticipate that CottonFGD will help to provide much useful information that should greatly facilitate efforts in cotton functional genomics research. CottonFGD also seems likely to play an important role in linking existent cotton-related database together, thus providing a comprehensive view of cotton genomics.

## Additional files

Additional file 1: List of all used cotton genome assemblies. Including seven cotton assemblies from four *Gossypium* species. (DOCX 23 kb)

Additional file 2: List of used RNA-seq data. Including 168 RNA-seq analyses for 20 experiment groups of four *Gossypium* species. (XLSX 36 kb)

Additional file 3: List of used WGS resequencing data. Including 96 analyses containing 79 *G. hirsutum* strains and 83 analyses containing 52 *G. barbadense* strains. (XLSX 31 kb)

Additional file 4: Snapshots of the search module. Several snapshots for the Browse page, the BLAST page and the Search page are provided. (PDF 1251 kb)

Additional file 5: Snapshots of the profile module. Several snapshots for the gene and transcript profile page are provided. (PDF 1306 kb)

Additional file 6: Snapshots of the analysis module. Several snapshots for the Analysis page, the Gene List Compare page and the phylogenetic tree build page are provided. (PDF 1094 kb)

## Abbreviations

BLAST: Basic Local Alignment Search Tool
BLAT: BLAST-Like Alignment Tool
BWA: Burrows-Wheeler Aligner
CDS: Coding DNA Sequence
DEG: Differential Expressed Gene
EMBOSS: European Molecular Biology Open Software Suite
FDR: False Discovery Rate
GFF: General Feature Format
GO: Gene Ontology
HTML5: HyperText Markup Language, version 5
INDEL: INsertion/DELetion
KAAS: KEGG Automatic Annotation Server
KEGG: Kyoto Encyclopedia of Genes and Genomes
MAFFT: Multiple Alignment using Fast Fourier Transform
MSA: Multiple Sequence Alignment
MySQL: My’s Structured Query Language
NCBI: National Center for Biotechnology Information
NGS QC Toolkit: Next-Generation Sequencing Quality Control Toolkit
PHP: PHP Hypertext Preprocessor
RFLP: Restricted Fragment Length Polymorphism
SNP: Single-Nucleotide Polymorphism
SRA: Sequence Read Archive
SSR: Simple Sequence Repeat
URL: Uniform Resource Locator
WGS: Whole Genome Shot-gun resequencing
