# Supplementary material for: CottonFGD: an integrated functional genomics database for cotton
Source: BMC Plant Biol. 2017 Jun 8;17:101. doi: 10.1186/s12870-017-1039-x (PMC5465443; doi:10.1186/s12870-017-1039-x)
Supplement: Supplementary file 1 — List of all used cotton genome assemblies. Including seven cotton assemblies from four Gossypium species. (DOCX 23 kb) [file 12870_2017_1039_MOESM1_ESM.docx]

Table S1-1. Seven available cotton genome assemblies.

| Species^*^ | Date Provider | Assembly Size (Mb) | Chromosome Number^**^ | Annotated Genes | Download Link |
| --- | --- | --- | --- | --- | --- |
| *G. raimondii*  (Ulbr.) | Joint Genome Institute (JGI)[1] | 761.4 | 13 (+1020) | 37505 | <http://phytozome.jgi.doe.gov/> |
| *G. raimondii*  (D_5_-3) | Beijing Genome Institute (BGI)[2] | 775.2 | 13 (+4434) | 40976 | <http://cgp.genomics.org.cn> |
| *G. arboretum*  (Shixiya1) | Beijing Genome Institute (BGI)[3] | 1694.6 | 13 (+75581) | 41331 | <http://cgp.genomics.org.cn> |
| *G. hirsutum*  (Tm-1) | Nanjing Agricultural University (NAU)[4] | 2447.0 | 26 (+38951) | 70478 | <http://mascotton.njau.edu.cn/html/Data/1.html> |
| *G. hirsutum*  (Tm-1) | Beijing Genome Institute (BGI)[5] | 2150.9 | 26 (+9141) | 76943 | <http://cgp.genomics.org.cn> |
| *G. barbadense*  (Xinhai-21) | Nanjing Agricultural University (NAU)[6] | 2263.5 | 26 (+2013) | 77358 | <http://database.chgc.sh.cn/cotton/index.html> |
| *G. barbadense*  (3-79) | Huazhong Agricultural University (HAU)[7] | 2573.2 | 26 (+17460) | 80876 | <http://cotton.cropdb.org/cotton/index.php> |

^*^Sequenced strains are listed in brackets.

^**^Unplaced scaffold numbers are listed in brackets

Table S1-2. Annotation consistence check.

| Assembly | No. proteins | No. CDS sequence consistent proteins | No. normal ORF  start/stop proteins | No. normal CDS length proteins |
| --- | --- | --- | --- | --- |
| *G. hirsutum*, NAU assembly | 70478 | 70478 (100%) | 68985 (97.9%) | 70478 (100%) |
| *G. hirsutum*, BGI assembly | 76943 | 75545 (98.2%) | 75572 (98.2%) | 76943 (100%) |
| *G. barbadense*, HAU assembly^*^ | 109918 | 21020 (19.1%) | 18858 (17.2%) | 103932 (94.5%) |
| *G. barbadense*, NAU assembly | 77358 | 77358 (100%) | 77358 (100%) | 77358 (100%) |
| *G. raimondii*, JGI assembly | 77267 | 77267 (100%) | 75666 (97.9%) | 77267 (100%) |
| *G. raimondii*, BGI assembly | 40976 | 40976 (100%) | 40638 (99.2%) | 40976 (100%) |
| *G. arboretum*, BGI assembly | 41331 | 41331 (100%) | 40246 (97.4%) | 41331 (100%) |

^*^For each assembly, we extracted all the CDS sequences based on its GFF annotation file and assembly sequences from data providers. Then the extracted CDS sequences were compared with provided CDS sequence file. The results showed that except for the HAU assembly of *G. barbadense*, all other assemblies reached ~100% consistence. In addition, we also measured whether the extracted CDS sequences have normal start/stop codons and normal length (3n). All assemblies had nearly 100% normal CDS length, but only CDS sequences from *G. barbadense*, HAU assembly had few normal start/stop codons. Therefore, the provided *G. barbadense* HAU assembly sequence from[7] (and also from CottonGen[8]) might have errors in scaffold orientation.

Table S3. Changes to the original annotations from data providers.

a) Chromosome ID.

| Assembly | Original ID | Changed ID |
| --- | --- | --- |
| *G. hirsutum*, NAU assembly | A01~A13;  D01~D13 | A01~A13;  D01~D13 (Unchanged) |
| *G. hirsutum*, BGI assembly | At_chr1~At_chr13;  Dt_chr1~Dt_chr13; | A01~A13;  D01~D13 |
| *G. barbadense*, NAU assembly | A01~A13;  D01~D13 | A01~A13;  D01~D13 (Unchanged) |
| *G. raimondii*, JGI assembly | Chr01 ~ Chr13 | Chr01 ~ Chr13 (Unchanged) |
| *G. raimondii*, BGI assembly | Chr1 ~ Chr13 | Chr01 ~ Chr13 |
| *G. arboretum*, BGI assembly | Chr1 ~ Chr13(CottonGen)  CA_chr1 ~ CA_chr13 (BGI) | Chr01 ~ Chr13 |

Chromosome IDs of diploids (*G. raimondii* and *G. arboretum*) were renamed as “Chr01 ~ Chr13”, while IDs of polyploids (*G. hirsutum* and *G. barbadense*) were renamed as “A01~A13, D01~D13”.

b) Transcript IDs.

Except for *G. raimondii* JGI assembly, all the other assemblies did not have alternative spliced isoforms annotated. Therefore, each transcript shared the same ID with its parental gene. In order to distinguish from transcript IDs from gene IDs, we append code “.1” to the gene IDs as their new transcript IDs. In the future, possible alternative isoforms could be added as “.2”, “.3”, …

**References**

1. Paterson AH, Wendel JF, Gundlach H, Guo H, Jenkins J, Jin D, Llewellyn D, Showmaker KC, Shu S, Udall J: **Repeated polyploidization of Gossypium genomes and the evolution of spinnable cotton fibres**. *Nature* 2012, **492**(7429):423-427.

2. Wang K, Wang Z, Li F, Ye W, Wang J, Song G, Yue Z, Cong L, Shang H, Zhu S: **The draft genome of a diploid cotton Gossypium raimondii**. *Nat Genet* 2012, **44**(10):1098-1103.

3. Li F, Fan G, Wang K, Sun F, Yuan Y, Song G, Li Q, Ma Z, Lu C, Zou C: **Genome sequence of the cultivated cotton Gossypium arboreum**. *Nat Genet* 2014, **46**(6):567-572.

4. Zhang T, Hu Y, Jiang W, Fang L, Guan X, Chen J, Zhang J, Saski CA, Scheffler BE, Stelly DM: **Sequencing of allotetraploid cotton (Gossypium hirsutum L. acc. TM-1) provides a resource for fiber improvement**. *Nat Biotechnol* 2015, **33**(5):531-537.

5. Li F, Fan G, Lu C, Xiao G, Zou C, Kohel RJ, Ma Z, Shang H, Ma X, Wu J: **Genome sequence of cultivated Upland cotton (Gossypium hirsutum TM-1) provides insights into genome evolution**. *Nat Biotechnol* 2015, **33**(5):524-530.

6. Liu X, Zhao B, Zheng H-J, Hu Y, Lu G, Yang C-Q, Chen J-D, Chen J-J, Chen D-Y, Zhang L: ***Gossypium barbadense* genome sequence provides insight into the evolution of extra-long staple fiber and specialized metabolites**. *Scientific Reports* 2015, **5**.

7. Yuan D, Tang Z, Wang M, Gao W, Tu L, Jin X, Chen L, He Y, Zhang L, Zhu L: **The genome sequence of Sea-Island cotton (Gossypium barbadense) provides insights into the allopolyploidization and development of superior spinnable fibres**. *Scientific reports* 2015, **5**.

8. Yu J, Jung S, Cheng C-H, Ficklin SP, Lee T, Zheng P, Jones D, Percy RG, Main D: **CottonGen: a genomics, genetics and breeding database for cotton research**. *Nucleic Acids Res* 2014, **42**(D1):D1229-D1236.
