# Supplementary material for: CottonFGD: an integrated functional genomics database for cotton
Source: BMC Plant Biol. 2017 Jun 8;17:101. doi: 10.1186/s12870-017-1039-x (PMC5465443; doi:10.1186/s12870-017-1039-x)
Supplement: Supplementary file 4 — Snapshots of the search module. Several snapshots for the Browse page, the BLAST page and the Search page are provided. (PDF 1251 kb) [file 12870_2017_1039_MOESM4_ESM.pdf]

Addition File 4: Snapshots of the search module

Figure S4-1: Snapshots of the Browse page: browse genes by genomic position.........2
Figure S4-2: Snapshots of the Browse page: browse genes by genetic marker.............4
Figure S4-3: Snapshots of the BLAST page..6
Figure S4-4: Snapshots of the Search page: search by gene ID/name............................7
Figure S4-5: Snapshots of the Search page: search by domain or function annotations. 9
Figure S4-6: Snapshots of the Search page: search by gene expression profiles. 11
References..12

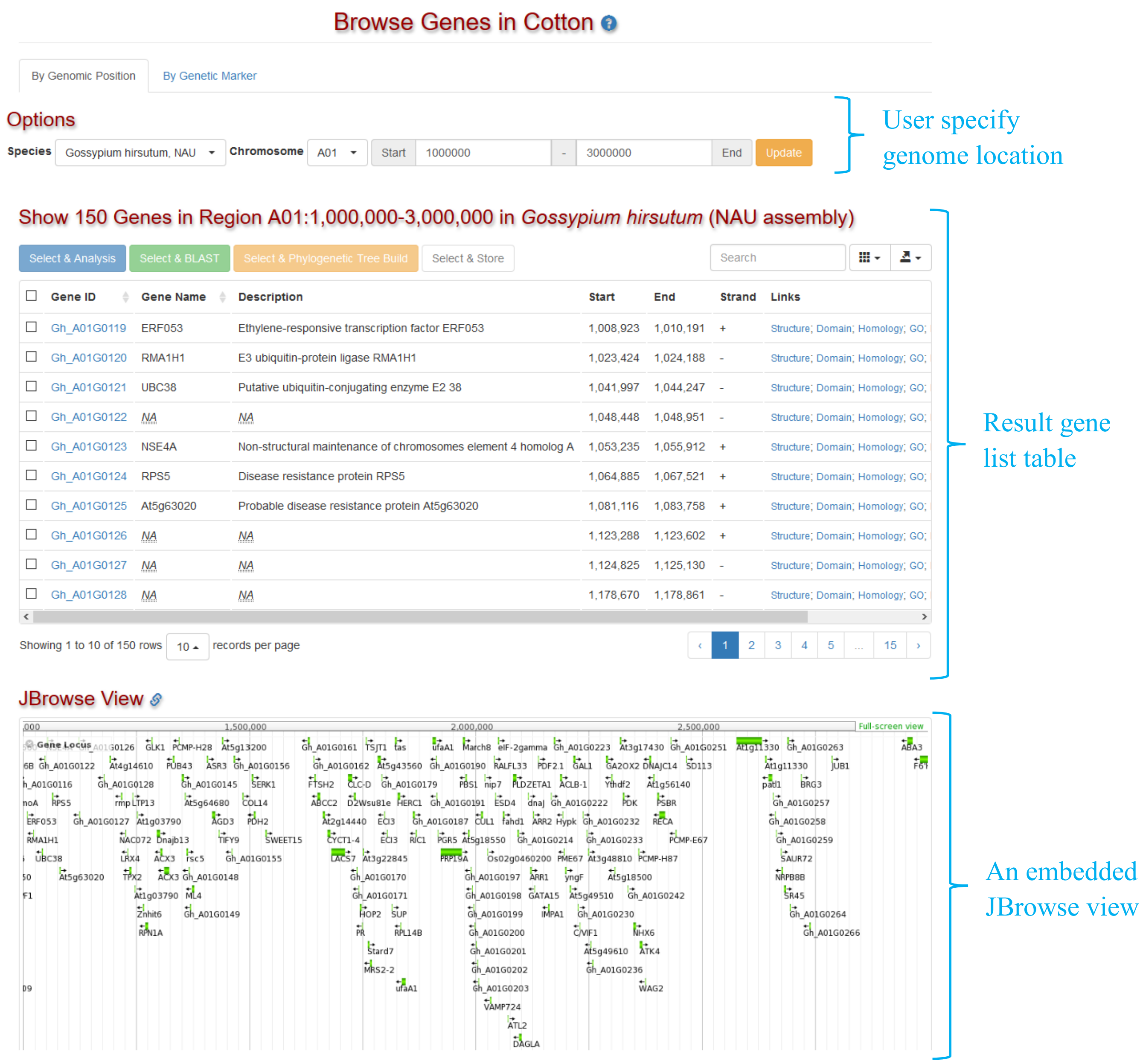

Figure S4-1: Snapshots of the Browse page: browse genes by genomic position. This snapshot displayed 150 genes in region A01:1,000,000-3,000,000 in *G. hirsutum* (NAU assembly), which was the default value when first visiting the Browse page.

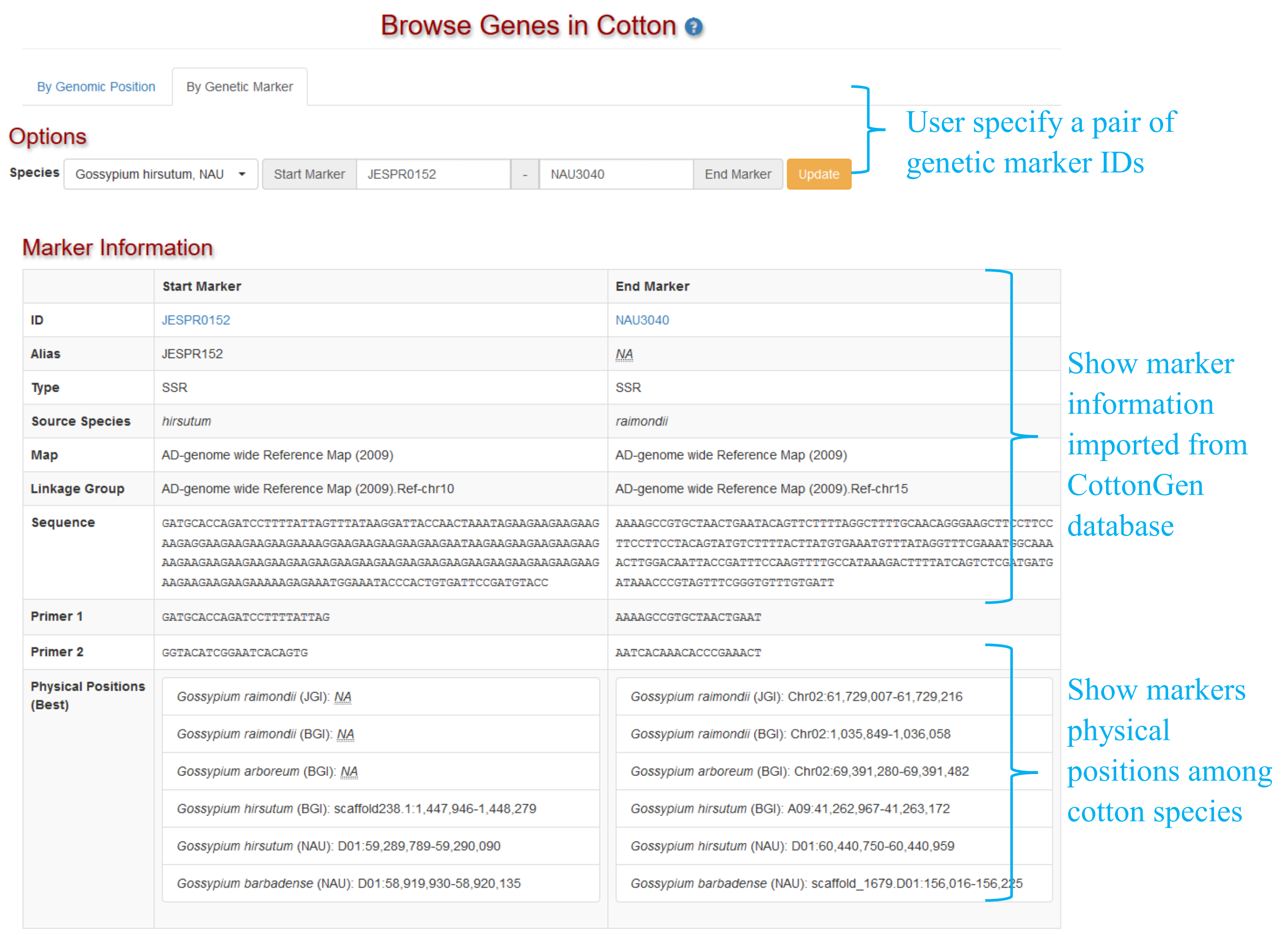
Browse Genes in Cotton

By Genomic Position | By Genetic Marker

Options

Species Gossypium hirsutum, NAU | Start Marker JESPR0152 - NAU3040 End Marker | Update

Marker Information

	Start Marker	End Marker
**ID**	JESPR0152	NAU3040
**Alias**	JESPR152	NA
**Type**	SSR	SSR
**Source Species**	*hirsutum*	*raimondii*
**Map**	AD-genome wide Reference Map (2009)	AD-genome wide Reference Map (2009)
**Linkage Group**	AD-genome wide Reference Map (2009).Ref-chr10	AD-genome wide Reference Map (2009).Ref-chr15
**Sequence**	GATGCACCAGATCCTTTTATTAGTTTATAAGGATTACCAACTAAATAGAAGAAGAAGAAG AAGAGGAAGAAGAAGAAGAAAAGGAAGAAGAAGAAGAATAAGAAGAAGAAGAAG AAGAAGAAGAAGAAGAAGAAGAAGAAGAAGAAGAAGAAGAAGAAGAAGAAGAAGAAG AAGAAGAAGAAGAAAAAGAGAAATGGAAATACCCACTGTGATTCCGATGTACC	AAAAGCCGTGCTAACTGAATACAGTTCTTTTAGGCTTTTGCAACAGGGAAGCTTCCTTCC TTCCTTCCTACAGTATGTCTTTTACTTATGTGAAATGTTTATAGGTTTCGAAATGGCAAA ACTTGGACAATTACCGATTTCCAAGTTTTGCCATAAAGACTTTTATCAGTCTCGATGATG ATAAACCCGTAGTTTCGGGTGTTTGTGATT
**Primer 1**	GATGCACCAGATCCTTTTATTAG	AAAAGCCGTGCTAACTGAAT
**Primer 2**	GGTACATCGGAATCACAGTG	AATCACAAACACCCGAAACT
**Physical Positions (Best)**	*Gossypium raimondii* (JGI): NA<br>*Gossypium raimondii* (BGI): NA<br>*Gossypium arboreum* (BGI): NA<br>*Gossypium hirsutum* (BGI): scaffold238.1:1,447,946-1,448,279<br>*Gossypium hirsutum* (NAU): D01:59,289,789-59,290,090<br>*Gossypium barbadense* (NAU): D01:58,919,930-58,920,135	*Gossypium raimondii* (JGI): Chr02:61,729,007-61,729,216<br>*Gossypium raimondii* (BGI): Chr02:1,035,849-1,036,058<br>*Gossypium arboreum* (BGI): Chr02:69,391,280-69,391,482<br>*Gossypium hirsutum* (BGI): A09:41,262,967-41,263,172<br>*Gossypium hirsutum* (NAU): D01:60,440,750-60,440,959<br>*Gossypium barbadense* (NAU): scaffold_1679.D01:156,016-156,225

Continue on the next page…

Continue the previous page …

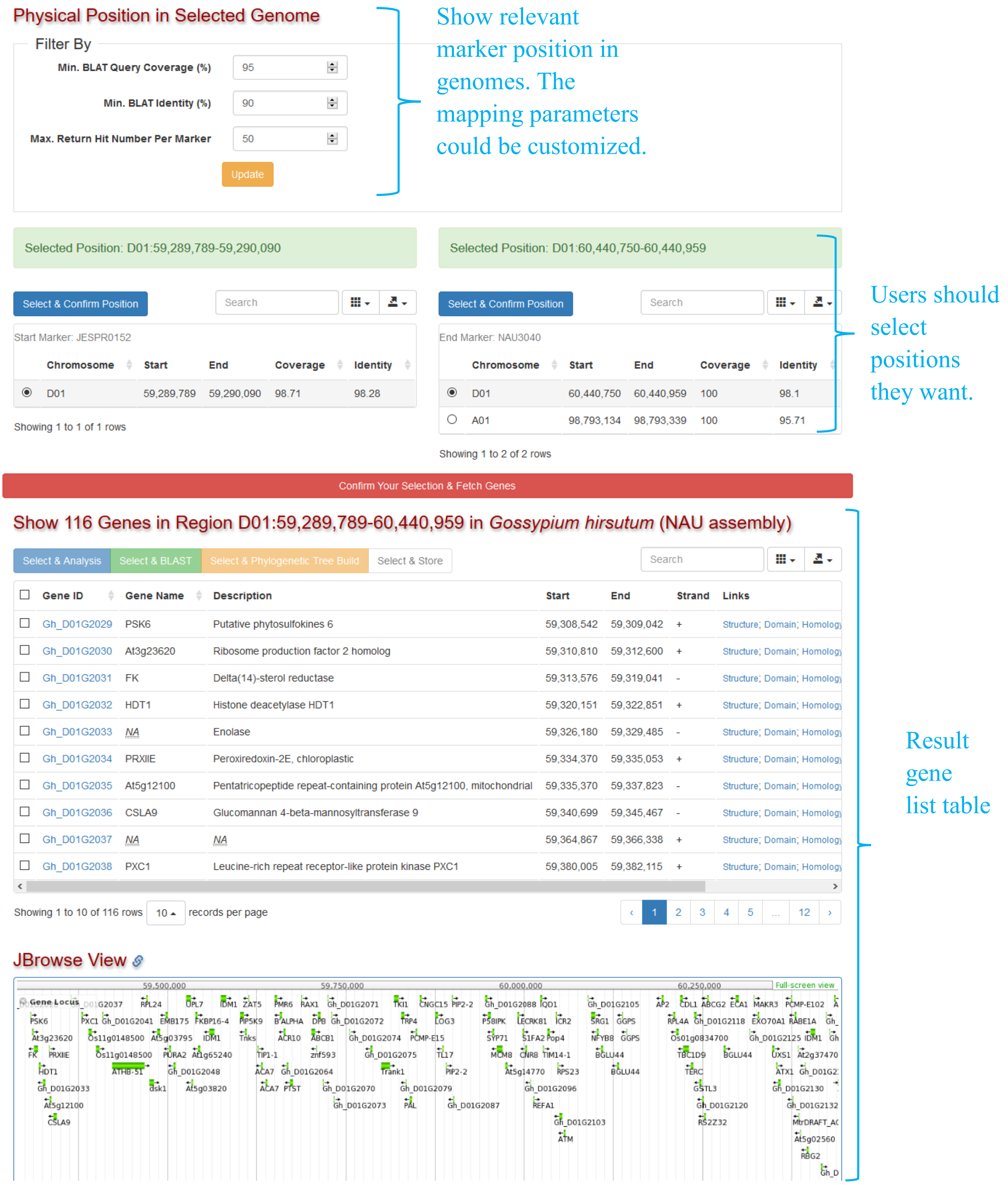

Figure S4-2: Snapshots of the Browse page: browse genes by genetic marker. Since a single marker might map to multiple genomic locations, CottonFGD first displays all the possible mapped genomic locations to let users select the positions they want. By default, CottonFGD displays genomic locations that have ≥95% query coverage and ≥90% identity, which could be customized by users. Then CottonFGD

displays the result gene list table based on users' selections. This snapshot displayed 116 *G. hirsutum* genes (NAU assembly) between SSR marker JESPR0152 and NAU3040.

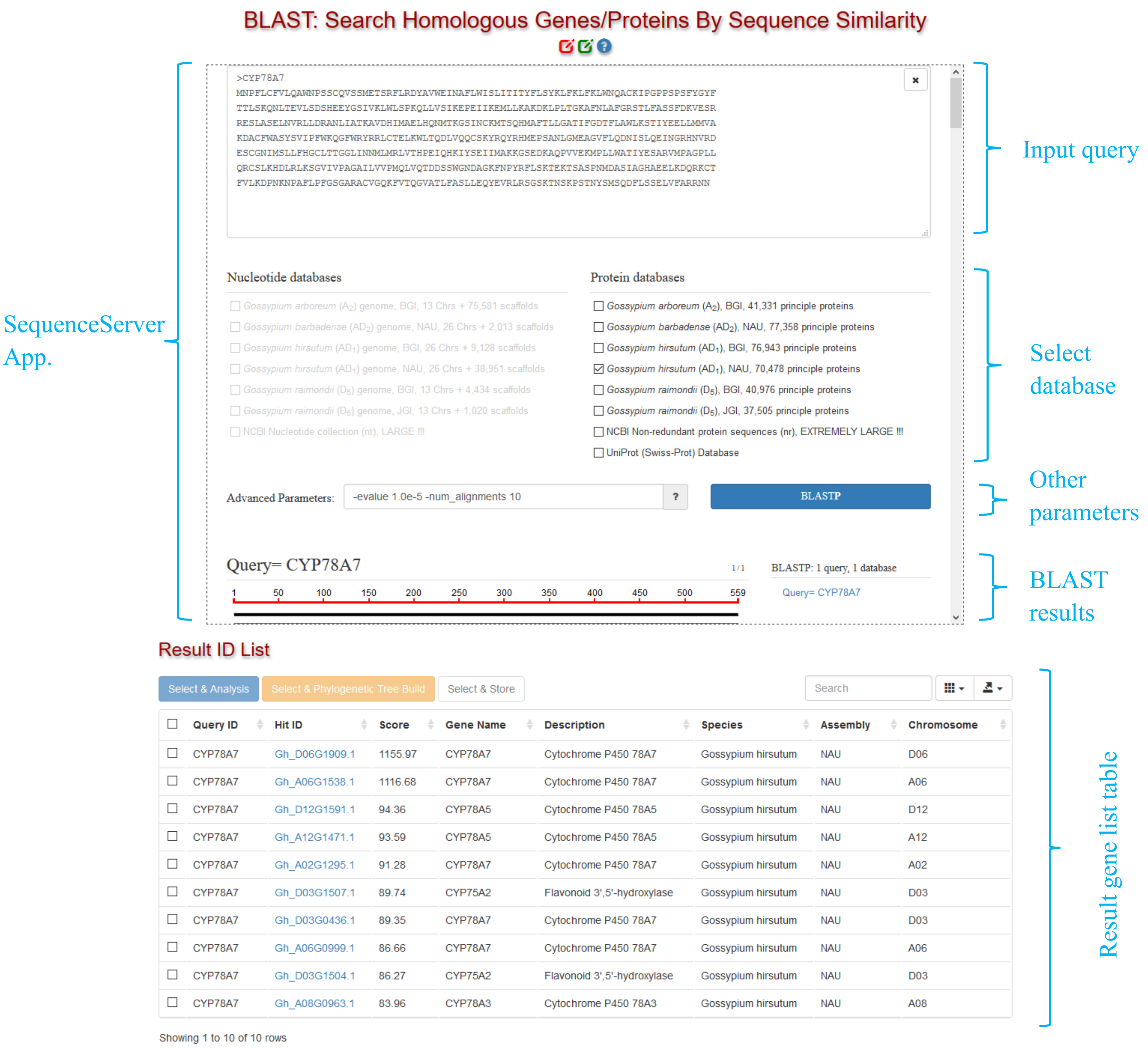

Figure S4-3: Snapshots of the BLAST page. This page mainly contains an embed SequenceServer App[1], a powerful BLAST front-end. After finishing the BLAST search, CottonFGD would automatically extract target gene IDs for each query sequence and rendered them in a table. This snapshot displayed the BLAST result using protein sequences of *CYP78A7* as query to search against all the *G. hirsutum* proteins with an E-value of 1e-05 and returning top 10 hits.

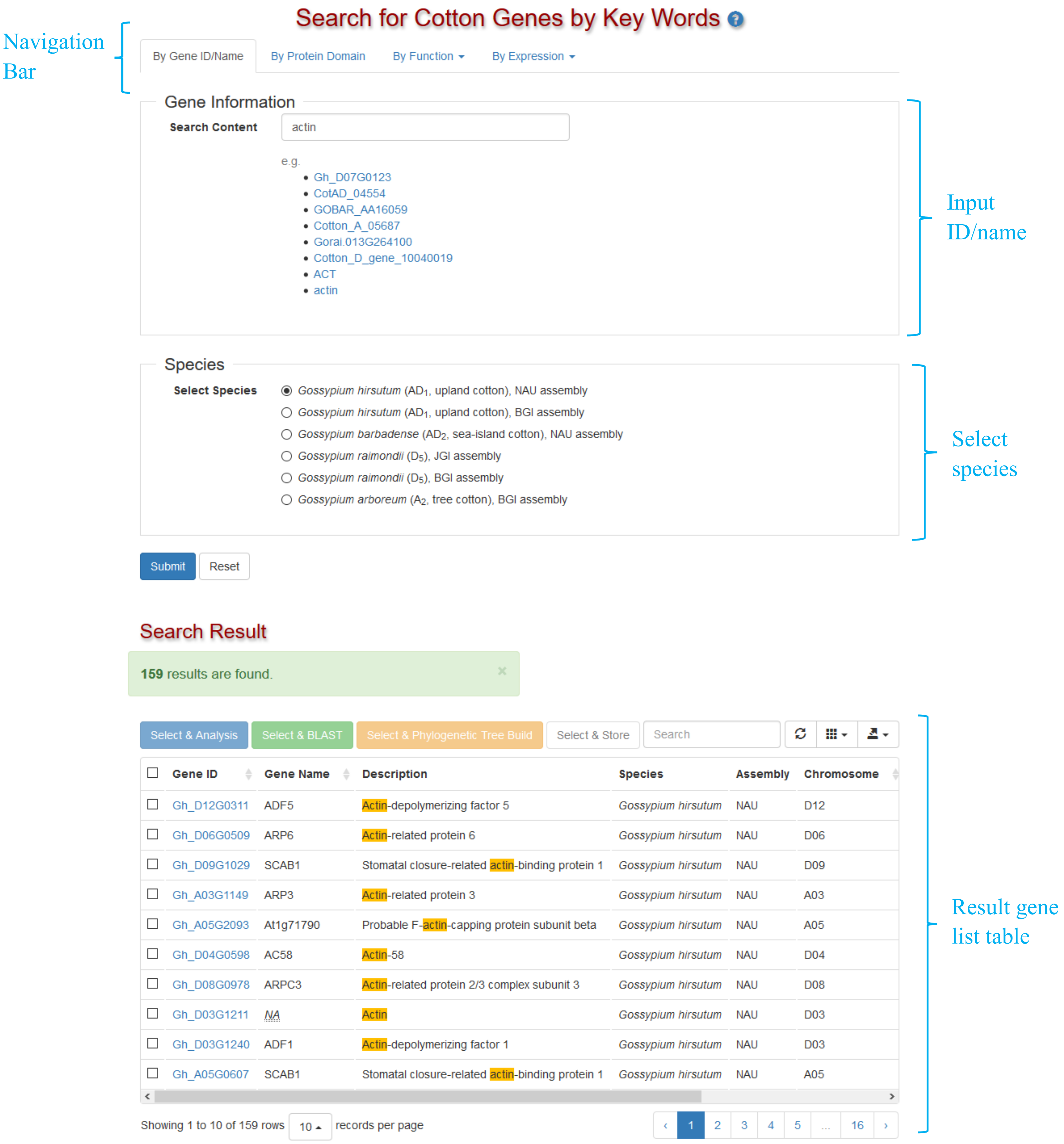

Figure S4-4: Snapshots of the Search page: search by gene ID/name. This snapshot displayed 159 *G. hirsutum* genes using keyword "actin" as gene name search.

Search for Cotton Genes by Key Words

By Gene ID/Name | By Protein Domain | By Function | By Expression

Domain Information

Domain Database: Pfam

Search Content: Thioesterase

e.g.
- PF03061
- PS00036
- 4HBT
- Thioesterase

Input domain ID/name (might be fuzzy)

Species

Select Species
- (selected) *Gossypium hirsutum* (AD_1, upland cotton), NAU assembly
- *Gossypium hirsutum* (AD_1, upland cotton), BGI assembly
- *Gossypium barbadense* (AD_2, sea-island cotton), NAU assembly
- *Gossypium raimondii* (D_5), JGI assembly
- *Gossypium raimondii* (D_5), BGI assembly
- *Gossypium arboreum* (A_2, tree cotton), BGI assembly

Select species

Submit | Reset

Search Result

Domain List

Select & Fetch Gene | Search

☐	Domain Accession	Domain Description	Exist in Cotton?
☐	PF00975	Thioesterase; Thioesterase domain	No
☐	PF01643	Acyl-ACP_TE; Acyl-ACP thioesterase	Yes
☐	PF02089	Palm_thioest; Palmitoyl protein thioesterase	Yes
☐	PF02551	Acyl_CoA_thio; Acyl-CoA thioesterase	No
☑	PF03061	4HBT; Thioesterase superfamily	Yes
☐	PF09500	YiiD_C; Putative thioesterase (yiiD_Cterm)	No
☐	PF10862	FcoT; FcoT-like thioesterase domain	No
☐	PF12590	Acyl-thio_N; Acyl-ATP thioesterase	Yes
☑	PF13279	4HBT_2; Thioesterase-like superfamily	Yes
☑	PF13622	4HBT_3; Thioesterase-like superfamily	Yes

Showing 1 to 10 of 10 rows

Step 1: Display all possible domains in selected domain database (e.g. Pfam) Users would select their wished items and perform a secondary search.

Search Result

Associated Proteins List

30 results are found.

Select & Analysis | Select & BLAST | Select & Phylogenetic Tree Build | Select & Store | Search

☐	Protein ID	Gene Name	Species	Assembly	Domain Accession
☐	Gh_A02G0603.1	DHNAT1	*Gossypium hirsutum*	NAU	PF03061 (1);
☐	Gh_A03G0409.1	ACOT13	*Gossypium hirsutum*	NAU	PF03061 (1);
☐	Gh_A04G0840.1	sll0410	*Gossypium hirsutum*	NAU	PF03061 (1);
☐	Gh_A04G0841.1	NA	*Gossypium hirsutum*	NAU	PF03061 (1);
☐	Gh_A05G0760.1	ACOT13	*Gossypium hirsutum*	NAU	PF03061 (1);
☐	Gh_A05G3848.1	ACOT13	*Gossypium hirsutum*	NAU	PF03061 (1);
☐	Gh_A08G1159.1	ACOT13	*Gossypium hirsutum*	NAU	PF03061 (1);
☐	Gh_A09G0781.1	Ascc1	*Gossypium hirsutum*	NAU	PF03061 (2);
☐	Gh_A09G0782.1	NA	*Gossypium hirsutum*	NAU	PF03061 (1);
☐	Gh_A09G0783.1	NA	*Gossypium hirsutum*	NAU	PF03061 (2);

Showing 1 to 10 of 30 rows | 10 | records per page | ‹ 1 2 3 ›

Step 2: Result gene list table based on users' selection

Figure S4-5: Snapshots of the Search page: search by domain or function annotations. As name match might be fuzzy, CottonFGD uses a two-step search method. This snapshot displayed searching *G. hirsutum* genes containing "Thioesterase" domain. The same method is also used for function (GO/InterPro/pathway) search.

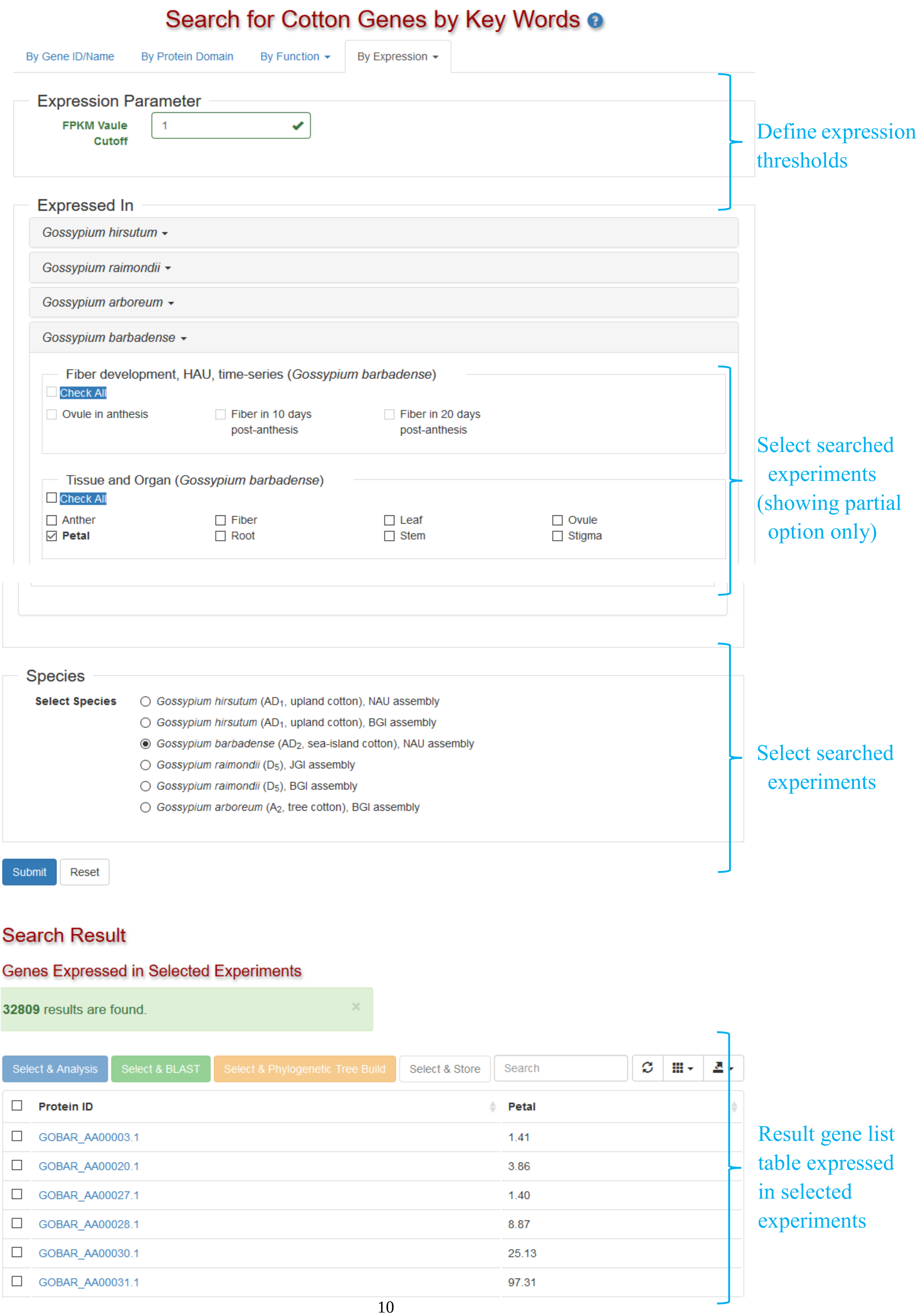
Search for Cotton Genes by Key Words
By Gene ID/Name
By Protein Domain
By Function
By Expression
Expression Parameter
FPKM Vaule Cutoff
1
Expressed In
Gossypium hirsutum
Gossypium raimondii
Gossypium arboreum
Gossypium barbadense
Fiber development, HAU, time-series (Gossypium barbadense)
Check All
Ovule in anthesis
Fiber in 10 days post-anthesis
Fiber in 20 days post-anthesis
Tissue and Organ (Gossypium barbadense)
Check All
Anther
Fiber
Leaf
Ovule
Petal
Root
Stem
Stigma
Species
Select Species
Gossypium hirsutum (AD1, upland cotton), NAU assembly
Gossypium hirsutum (AD1, upland cotton), BGI assembly
Gossypium barbadense (AD2, sea-island cotton), NAU assembly
Gossypium raimondii (D5), JGI assembly
Gossypium raimondii (D5), BGI assembly
Gossypium arboreum (A2, tree cotton), BGI assembly
Submit
Reset
Search Result
Genes Expressed in Selected Experiments
32809 results are found.
Select & Analysis
Select & BLAST
Select & Phylogenetic Tree Build
Select & Store
Search
Protein ID
Petal
GOBAR_AA00003.1
1.41
GOBAR_AA00020.1
3.86
GOBAR_AA00027.1
1.40
GOBAR_AA00028.1
8.87
GOBAR_AA00030.1
25.13
GOBAR_AA00031.1
97.31
Define expression thresholds
Select searched experiments (showing partial option only)
Select searched experiments
Result gene list table expressed in selected experiments

Figure S4-6: Snapshots of the Search page: search by gene expression profiles. The expression FPKM threshold could be customized (default value is 1). CottonFGD would display a list of genes expressed in all the selected experiments. This snapshot displayed searching *G. barbadense* genes expressed in petal.

References

1. Priyam A, Woodcroft BJ, Rai V, Munagala A, Moghul I, Ter F, Gibbins MA, Moon H, Leonard G, Rumpf W: **Sequenceserver: a modern graphical user interface for custom BLAST databases**. *Biorxiv* 2015:033142.
