# Supplementary material for: CottonFGD: an integrated functional genomics database for cotton
Source: BMC Plant Biol. 2017 Jun 8;17:101. doi: 10.1186/s12870-017-1039-x (PMC5465443; doi:10.1186/s12870-017-1039-x)
Supplement: Supplementary file 5 — Snapshots of the profile module. Several snapshots for the gene and transcript profile page are provided. (PDF 1306 kb) [file 12870_2017_1039_MOESM5_ESM.pdf]

Addition File 5: Snapshots of the profile module

Figure S5-1. Snapshots of the gene profile page. 2
Figure S5-2. Snapshots of the transcript profile page: Basic information. 3
Figure S5-3. Snapshots of the transcript profile page: DNA/protein statistics. 4
Figure S5-4. Snapshots of the transcript profile page: Exon-intron structure. 5
Figure S5-5. Snapshots of the transcript profile page: Protein domain. 6
Figure S5-6. Snapshots of the transcript profile page: Homology. 7
Figure S5-7. Snapshots of the transcript profile page: Function. 8
Figure S5-8. Snapshots of the transcript profile page: Expression. 10
Figure S5-9. Snapshots of the transcript profile page: Variation 12

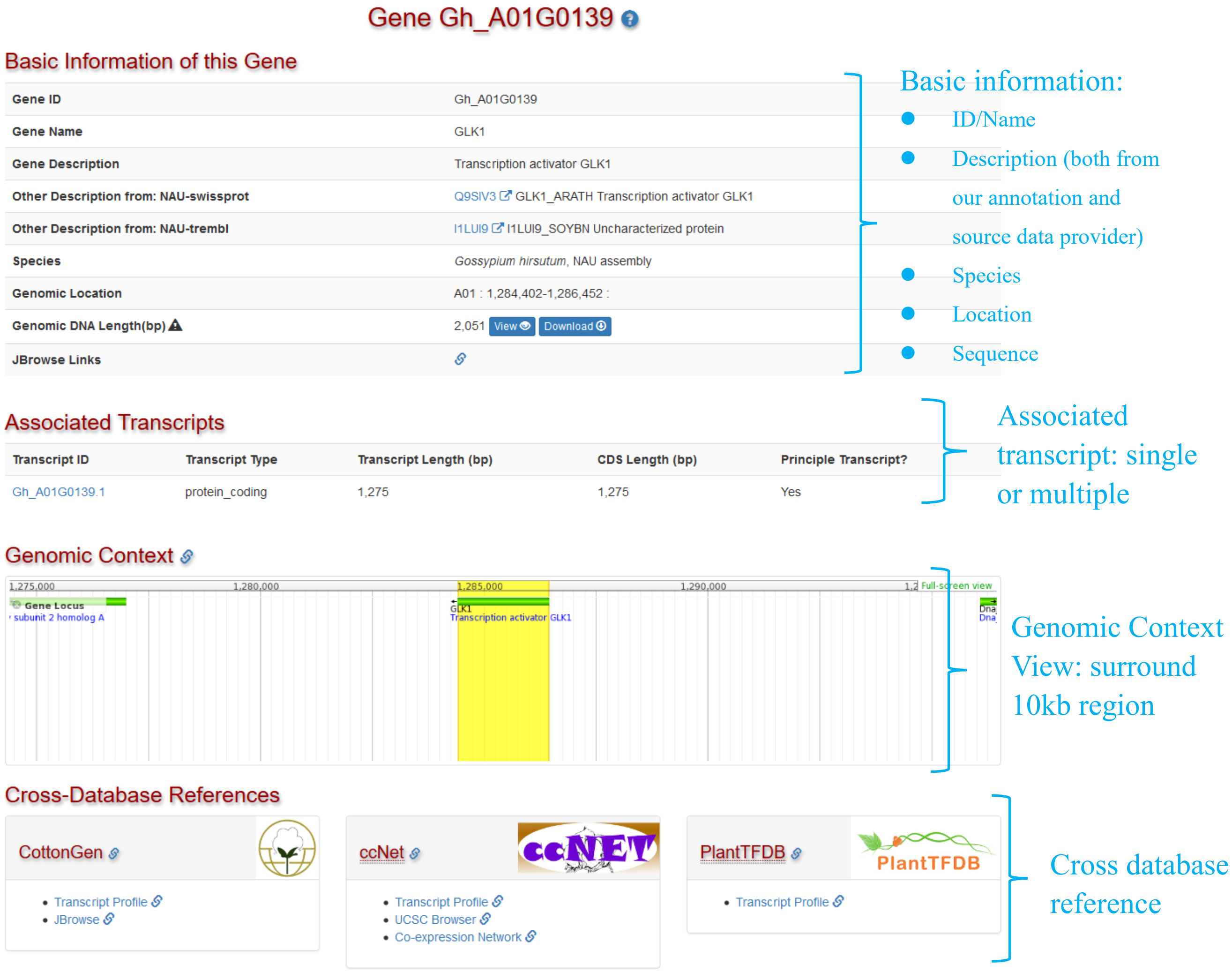

Figure S5-1. Snapshots of the gene profile page. This snapshot displayed profile pages of gene Gh_A01G0139 (*GLK1*) in *G. hirsutum* (NAU assembly).

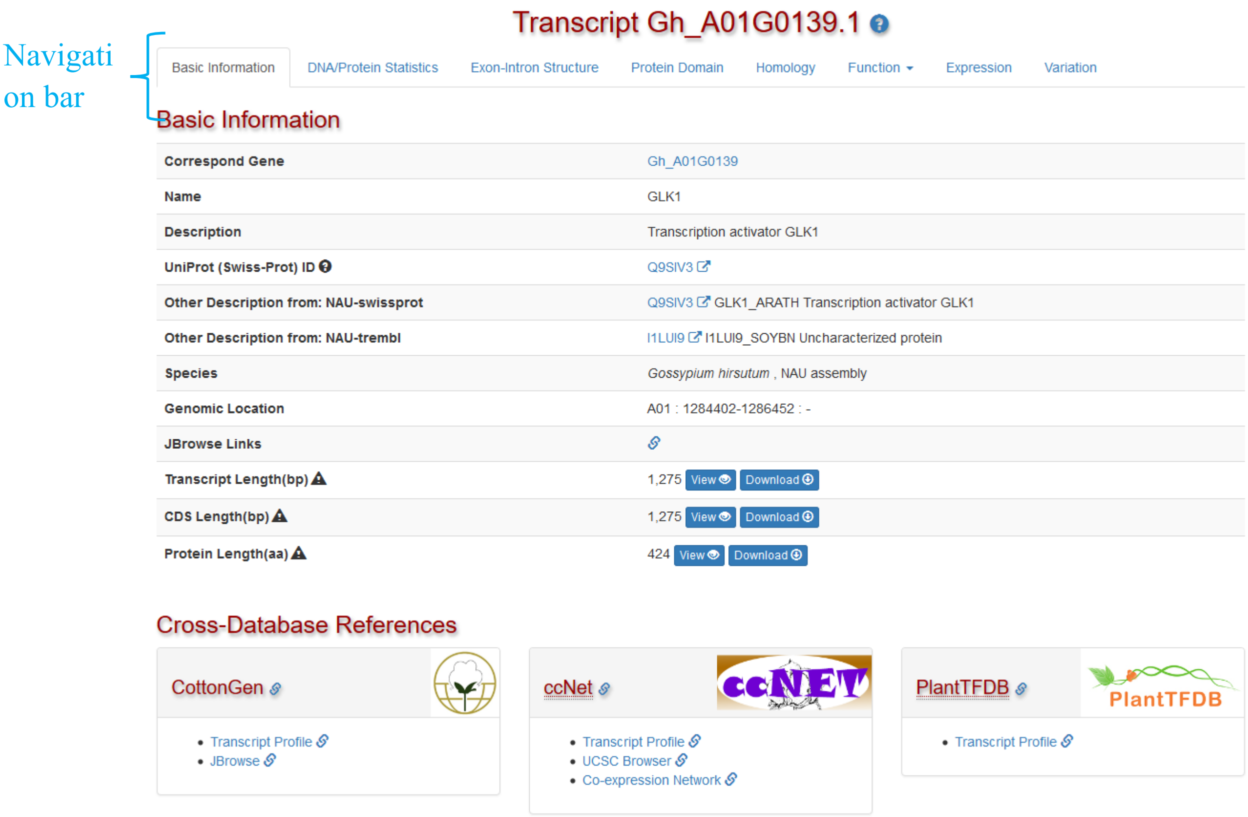

Figure S5-2. Snapshots of the transcript profile page: Basic information. This snapshot displayed ID/name, transcript/CDS/protein sequences and cross-database references of transcript Gh_A01G0139.1 (*GLK1*) in *G. hirsutum* (NAU assembly).

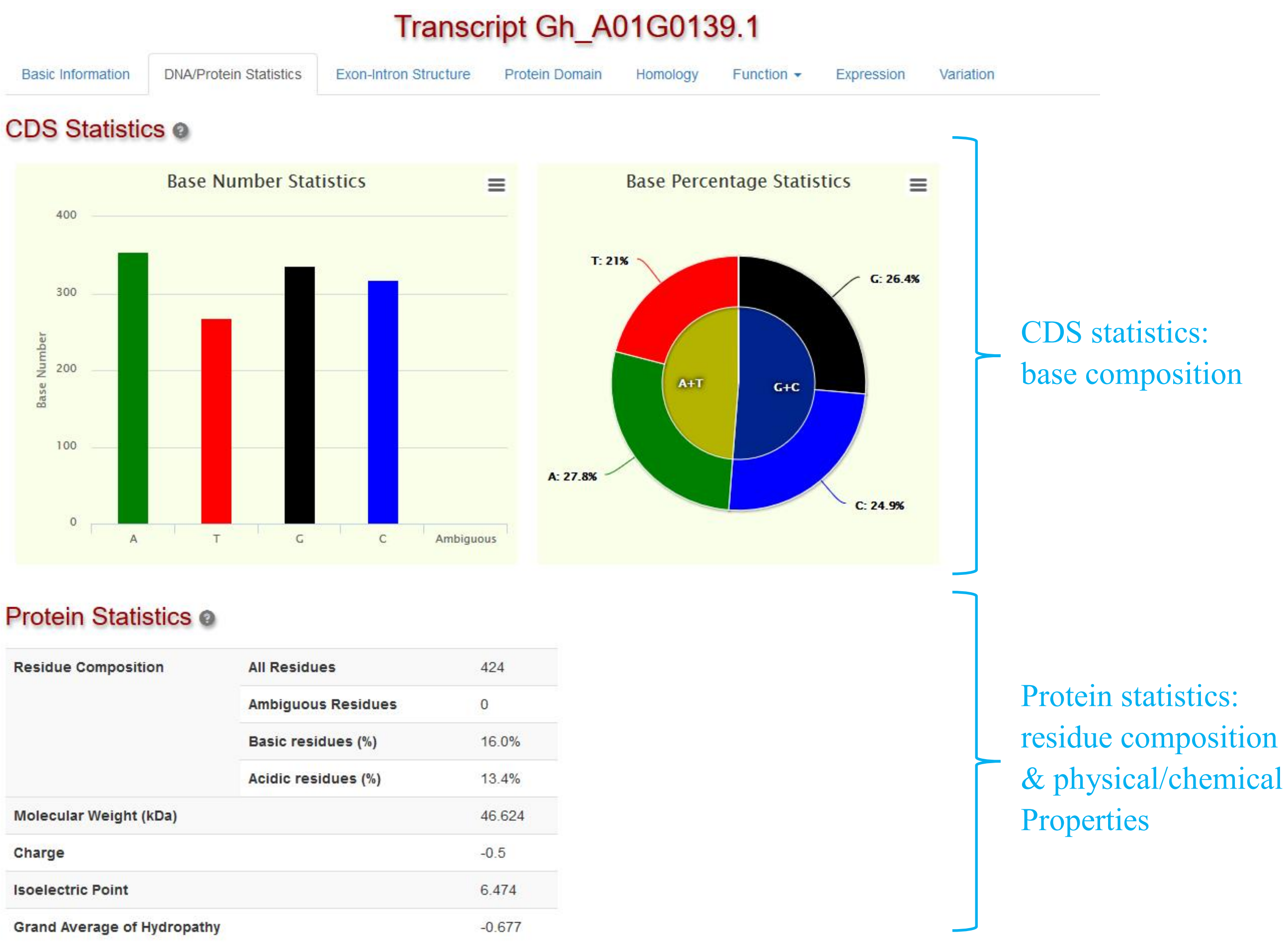

Figure S5-3. Snapshots of the transcript profile page: DNA/protein statistics. This snapshot displayed base composition, protein residue composition and protein physical/chemical properties of transcript Gh_A01G0139.1 (*GLK1*) in *G. hirsutum* (NAU assembly).

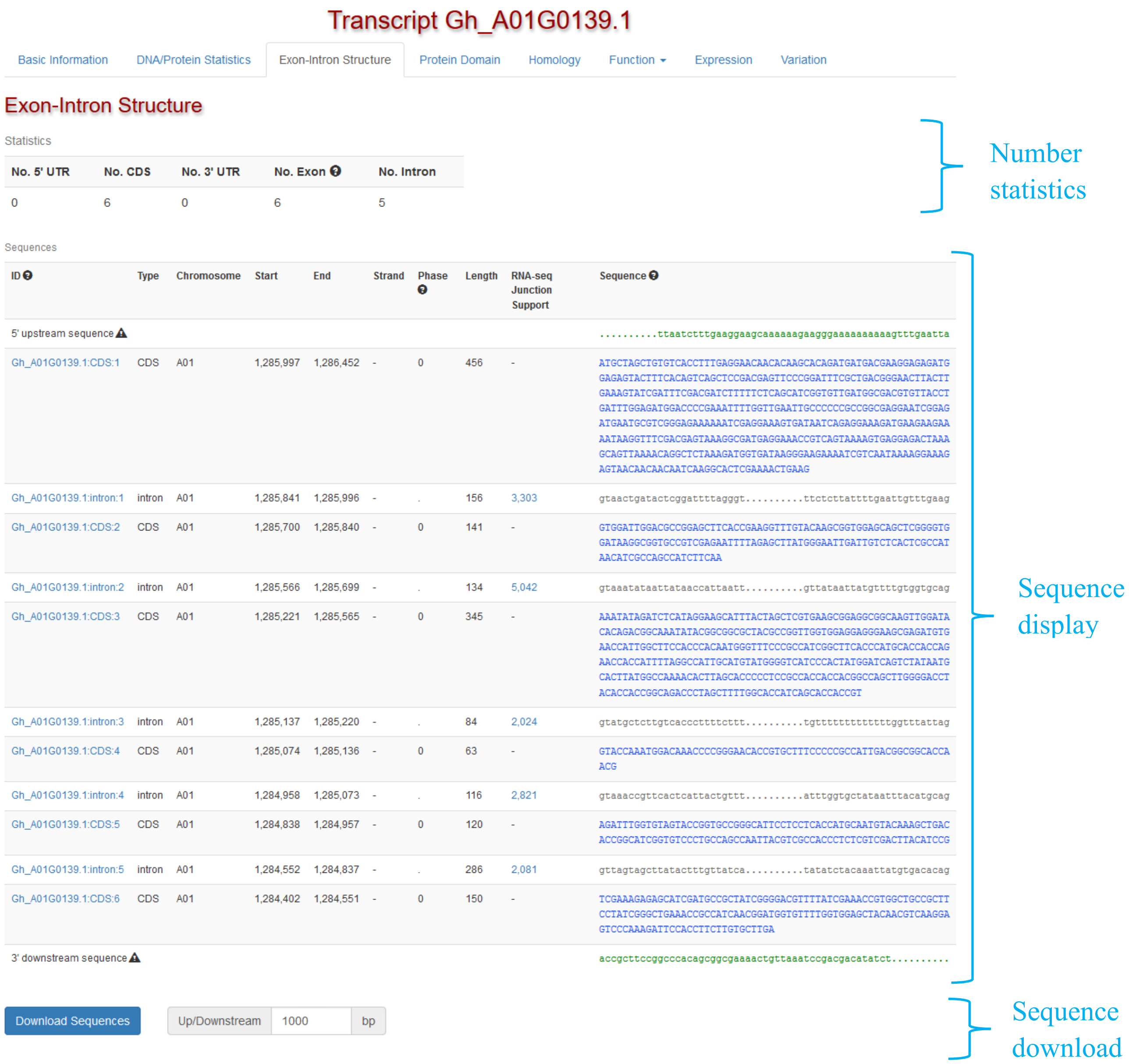

Figure S5-4. Snapshots of the transcript profile page: Exon-intron structure. This snapshot displayed all the sequences of each exon and intron in transcript Gh_A01G0139.1 (*GLK1*) in *G. hirsutum* (NAU assembly). Users can download these sequences together with upstream/downstream sequences in custom length.

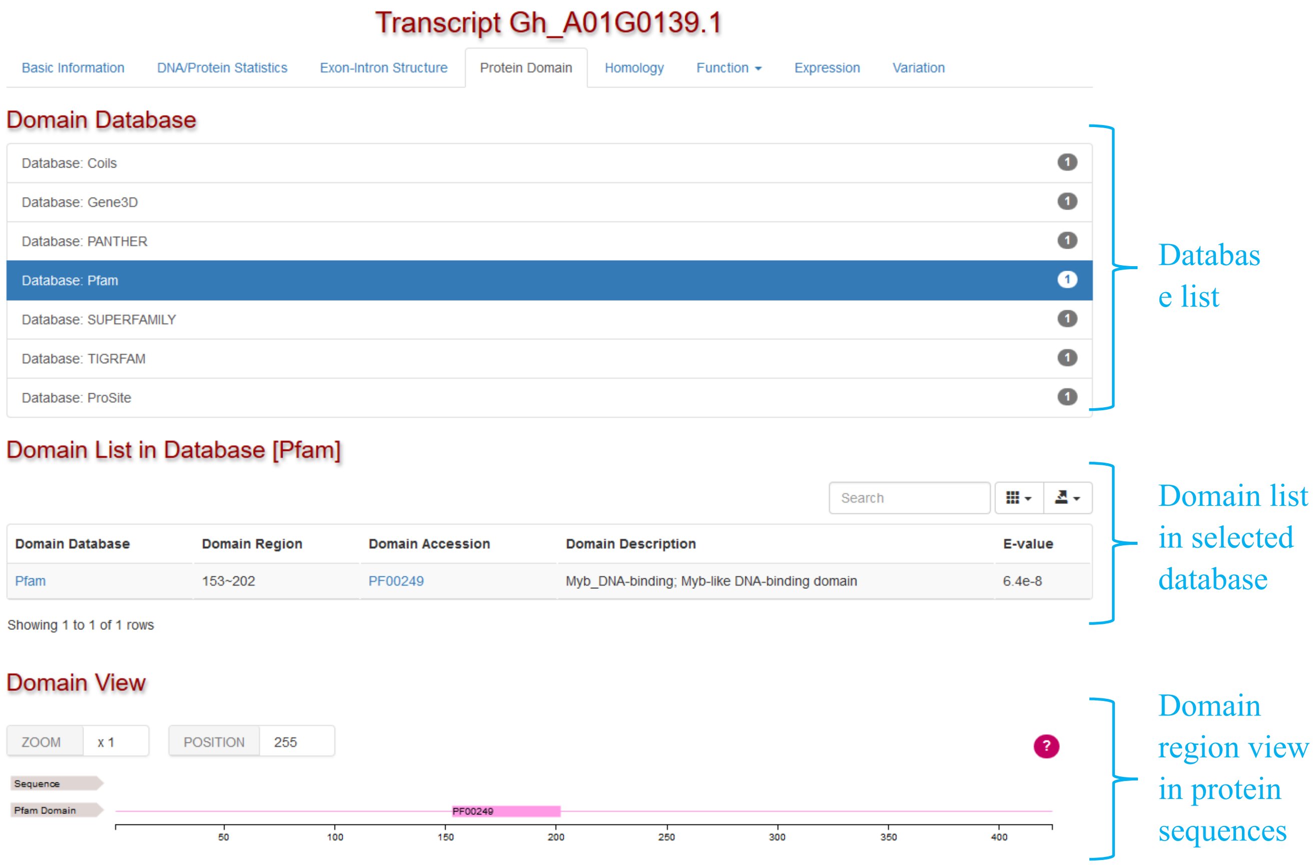

Figure S5-5. Snapshots of the transcript profile page: Protein domain. This snapshot displayed domains in protein Gh_A01G0139.1 (*GLK1*) in *G. hirsutum* (NAU assembly). Users can switch among different domain databases.

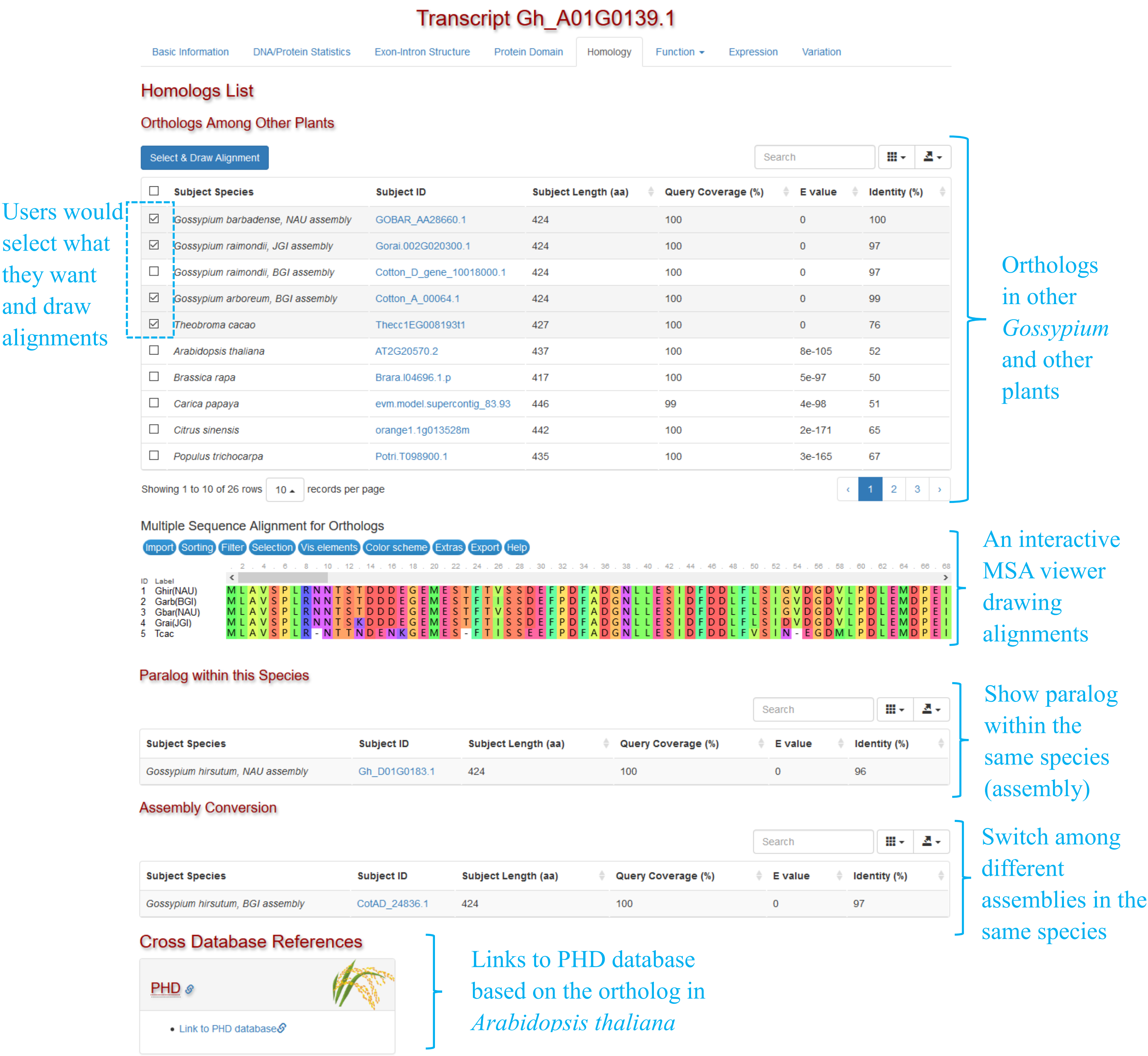

Figure S5-6. Snapshots of the transcript profile page: Homology. This snapshot displayed orthologs and paralogs in protein Gh_A01G0139.1 (*GLK1*) in *G. hirsutum* (NAU assembly). Users can select several target species and CottonFGD would draw an interactive alignment.

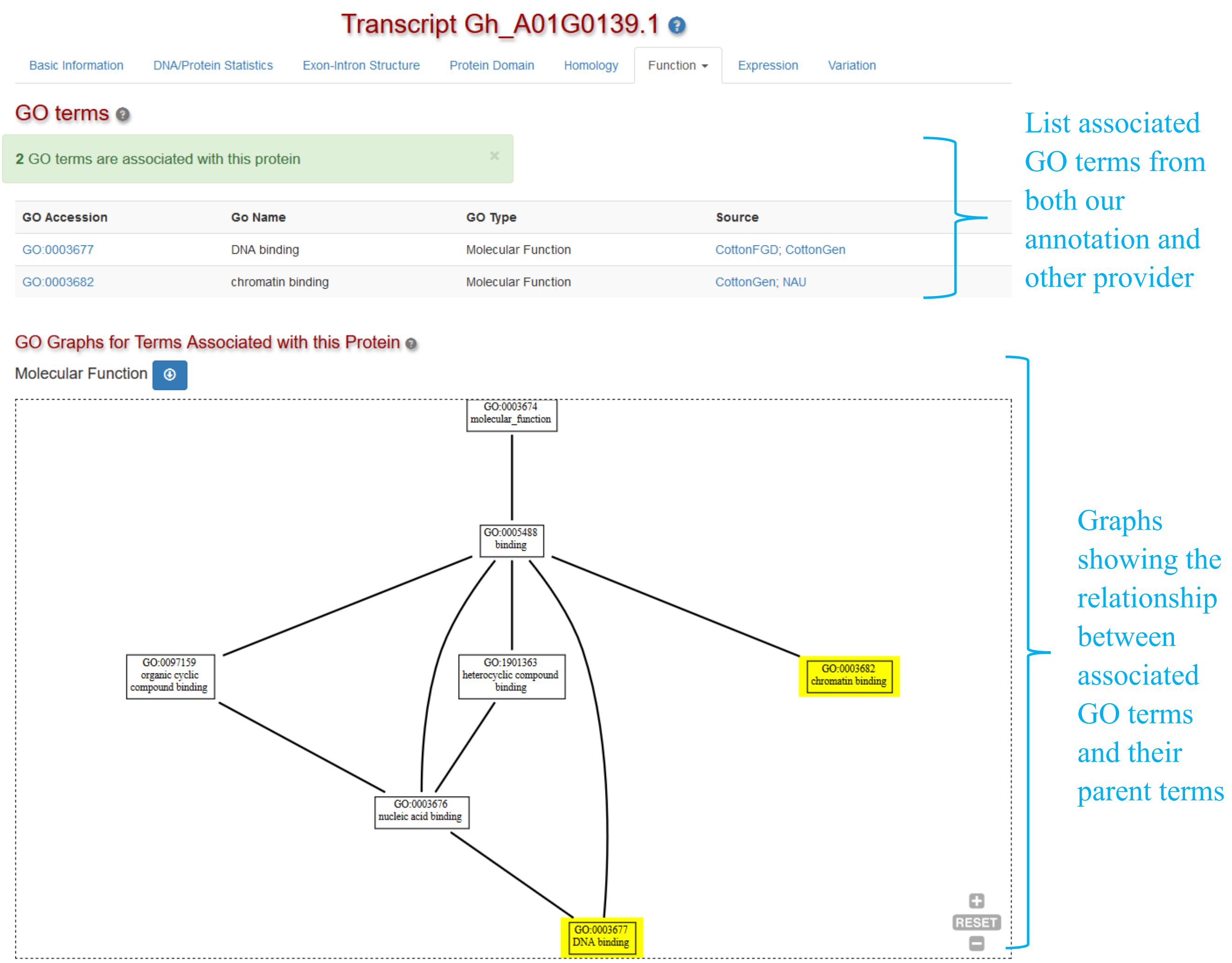

Figure S5-7. Snapshots of the transcript profile page: Function. This snapshot displayed associated GO terms in protein Gh_A01G0139.1 (*GLK1*) in *G. hirsutum* (NAU assembly). Interactive graphs showing the relationship between associated GO terms and their parent terms were also presented in three categories: Molecular Function, Biology Process and Cell Component. (This gene only has GO terms in "Molecular Function").

Transcript Gh_A01G0139.1

Basic Information | DNA/Protein Statistics | Exon-Intron Structure | Protein Domain | Homology | Function | Expression | Variation

Expression Groups

Experiment	Count
Cytoplasmic male sterility (CMS) study of strain JA and JB, sporogenous cell stage	1
Fiber development, time-series	2
Ovule development, time-series	3
Seed germination, cotyledon time-series	5
Seed germination, organ comparison	2
Seed germination, root time-series	2
Seed germination, seed time-series	3
Stress experiment, Cold treat in time-series	4
Stress experiment, compare with control	5
Stress experiment, Hot treat in time-series	4
Stress experiment, PEG treat in time-series	4
Stress experiment, Salt treat in time-series	4
Tissue and Organ	5

Experiment List Users would select what they want

[Stress experiment, Salt treat in time-series] Expression Levels

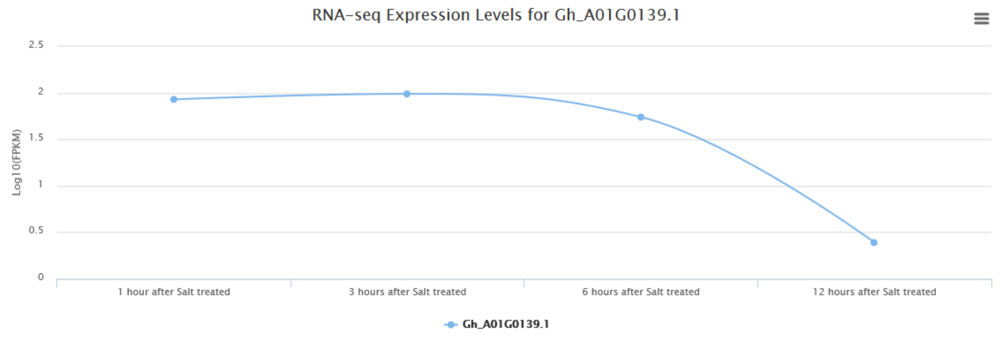

Search

Project	Run	Sample	FPKM
SRP044705	SRR1768512;	1 hour after Salt treated	84.27
SRP044705	SRR1768513;	3 hours after Salt treated	96.93
SRP044705	SRR1768514;	6 hours after Salt treated	55.27
SRP044705	SRR1768515;	12 hours after Salt treated	2.45

Showing 1 to 4 of 4 rows

Expression levels (FPKM) in selected experiment

[Stress experiment, Salt treat in time-series] Expression Difference

WARNING: **No** Significant Expression Difference in this group. ×

JBrowse View

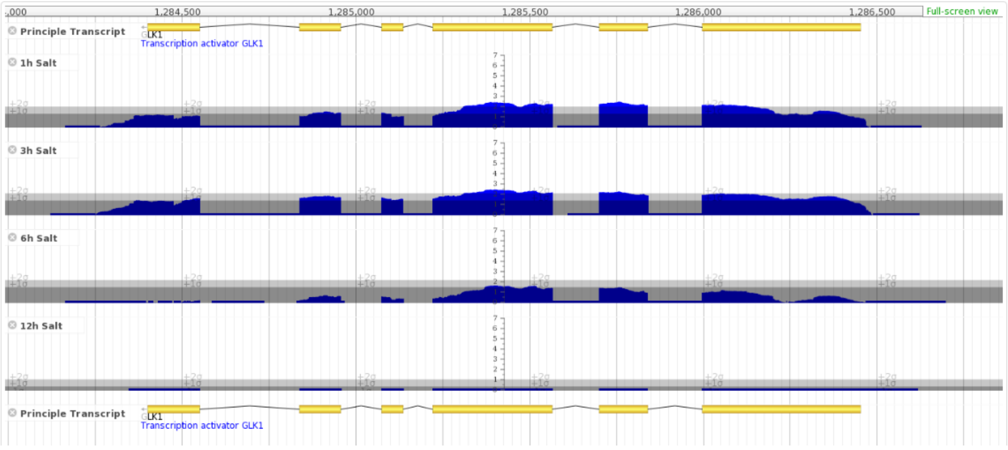

Visualization of RNA-seq coverages in selected experiment

Figure S5-8. Snapshots of the transcript profile page: Expression. This snapshot displayed the expression pattern of transcript Gh_A01G0139.1 (*GLK1*) in *G. hirsutum* (NAU assembly) in salt treated experiment compared in time-series (1h, 3h, 6h, 12h). CottonFGD would display a JBrowse snapshot of relevant RNA-seq coverages.

Transcript Gh_A01G0139.1

Basic Information | DNA/Protein Statistics | Exon-Intron Structure | Protein Domain | Homology | Function ▾ | Expression | Variation

Variation

SNP Sites

Refresh SNPs | Within Up/Downstream 2000 bp

Search

Position	Ref. Allele.	Alt. Allele.	Effect	Impact	M.A.F.
A01:1,287,949	G(127)	C(1)	upstream_gene_variant	MODIFIER	0.008
A01:1,287,803	T(139)	C(3)	upstream_gene_variant	MODIFIER	0.021
A01:1,287,496	G(115)	A(1)	upstream_gene_variant	MODIFIER	0.009
A01:1,287,460	C(110)	T(4)	upstream_gene_variant	MODIFIER	0.035
A01:1,287,229	A(108)	T(2)	upstream_gene_variant	MODIFIER	0.018
A01:1,286,002	G(151)	C(1)	missense_variant	MODERATE	0.007
A01:1,285,477	G(115)	T(1)	missense_variant	MODERATE	0.009
A01:1,285,473	C(117)	A(1)	synonymous_variant	LOW	0.008
A01:1,283,756	C(93)	T(1)	downstream_gene_variant	MODIFIER	0.011
A01:1,283,549	C(116)	T(2)	downstream_gene_variant	MODIFIER	0.017

Showing 1 to 10 of 13 rows 10 records per page ‹ 1 2 ›

Show SNPs within this transcript and in surround region. Users would custom the region length

Population Genetic Analysis

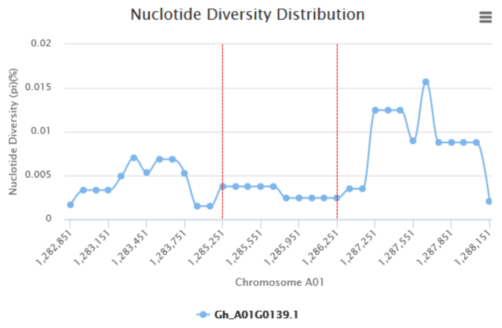

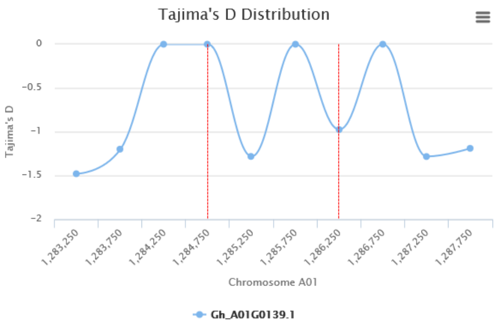

Parameter ▾

Window size: 500 bp

Step size: 100 bp

Filter Sites: ☐ Exclude sites containing heterozygous genotype

Select Sub-sample sets ▾

☐ Check All

☐ 108ASA	☐ 66-241	☐ 86-1	☐ Auburn_56
☐ Brazil_0101	☐ Brazil_011	☐ Brazil_014	☐ Brazil_cotton
☐ CIR10	☐ CIR12	☐ CIR35	☐ CIR7
☐ Coker's_Foster_300	☐ Coker139	☐ Delfos_531C	☐ Dixie_King
☐ Dixie_Triumph	☐ Esha_28	☐ Ferguson_406	☐ India_941
☐ India_NV50-70*	☐ J8891*	☐ Jimian_1#	☐ Junmian_1#
☐ King	☐ Lanart_57	☐ Lankart	☐ Latifolium_1
☐ Latifolium_13	☐ Latifolium_161	☐ Latifolium_178	☐ Latifolium_3
☐ Latifolium_39	☐ Latifolium_64	☐ Lone_Star	☐ Lumian_1#
☐ MA-6	☐ Marie-galante_17	☐ Marie-galante_36	☐ Marie-galante_62
☐ Marie-galante_7	☐ Marie-galante_71	☐ Mebane	☐ Mebane_Triumph
☐ Morrilli_2	☐ Morrilli_38	☐ Morrilli_45	☐ Palmeri_2
☐ Palmeri_5	☐ Paymaster_54	☐ Paymaster_54B	☐ Punctatum_12
☐ Punctatum_14	☐ Punctatum_24	☐ Punctatum_25	☐ Punctatum_3
☐ Punctatum_31	☐ Punctatum_5	☐ Richmondi_2	☐ Richmondi_3
☐ Richmondi_7	☐ Rowden_Malone	☐ Shiyuan_321	☐ Simian_2#
☐ Simian_3#	☐ Stoneville2B	☐ Sumian_12#	☐ Tashkent_1
☐ Tashkent_2	☐ Tashkent_3	☐ Trice	☐ Xinluzao_1#_(GK62)
☐ Xinluzao_26#	☐ Xinluzao_42#	☐ Yucatanense_1	☐ Yucatanense_2
☐ Yucatanense_3	☐ Yucatanense_5A	☐ Yucatanense_6	

Redraw Charts | Get Sample Genotype

Show several population genetic value calculated from the SNP data. Users would custom the window/step size and the samples.

Figure S5-9. Snapshots of the transcript profile page: Variation (currently we only implemented SNPs in *G. hirsutum* NAU assembly and *G. barbadense* NAU assembly). This snapshot displayed the SNPs of transcript Gh_A01G0139.1 (*GLK1*) in *G. hirsutum* (NAU assembly). Length of upstream/downstream regions, parameters of population genetics analysis (currently only nucleotide diversity and Tajima's D are implemented) and sample selection could be customized.
