# Supplementary material for: CottonFGD: an integrated functional genomics database for cotton
Source: BMC Plant Biol. 2017 Jun 8;17:101. doi: 10.1186/s12870-017-1039-x (PMC5465443; doi:10.1186/s12870-017-1039-x)
Supplement: Supplementary file 6 — Snapshots of the analysis module. Several snapshots for the Analysis page, the Gene List Compare page and the phylogenetic tree build page are provided. (PDF 1094 kb) [file 12870_2017_1039_MOESM6_ESM.pdf]

Addition File 6: Snapshots of the analysis module

Figure S6-1. Snapshots of the analysis page: fetch structure information....................2
Figure S6-2. Snapshots of the analysis page: fetch function information. 3
Figure S6-3. Snapshots of the analysis page: fetch expression level information.........5
Figure S6-4. Snapshots of the analysis page: enrichment analysis...............................7
Figure S6-5. Snapshots of the list compare page..9
Figure S6-6. Snapshots of the phylogenetic tree build page.......................................10

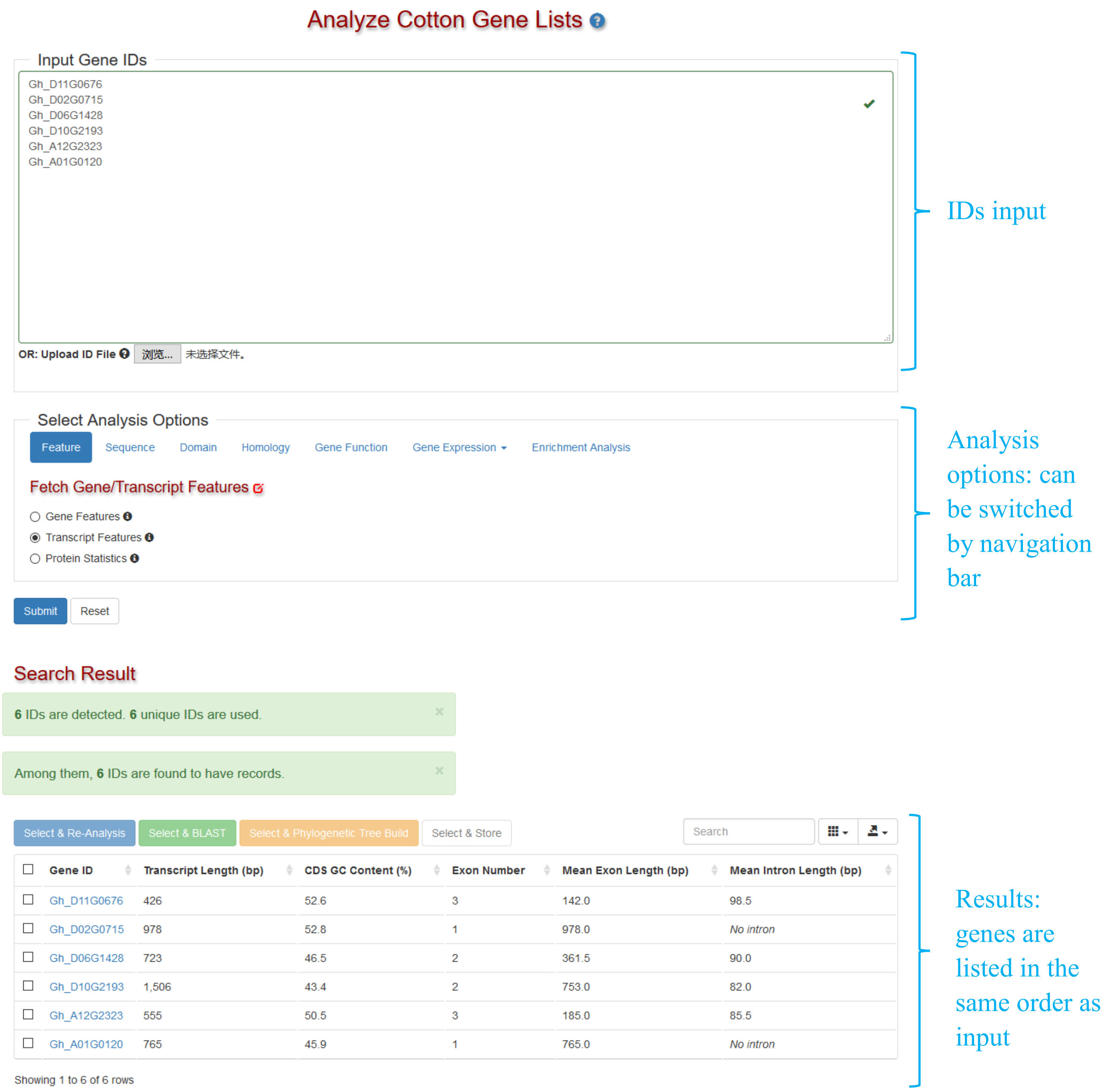

Figure S6-1. Snapshots of the analysis page: fetch structure information. This snapshot displayed fetching transcript structure information in six genes in *G. hirsutum* (NAU assembly). Genes are: Gh_D11G0676, Gh_D02G0715, Gh_D06G1428, Gh_D10G2193, Gh_A12G2323 and Gh_A01G0120. The fetching results are in exactly the same order as the inputted IDs. (Note: this snapshot hid several columns in the result table in order to show the main ones.)

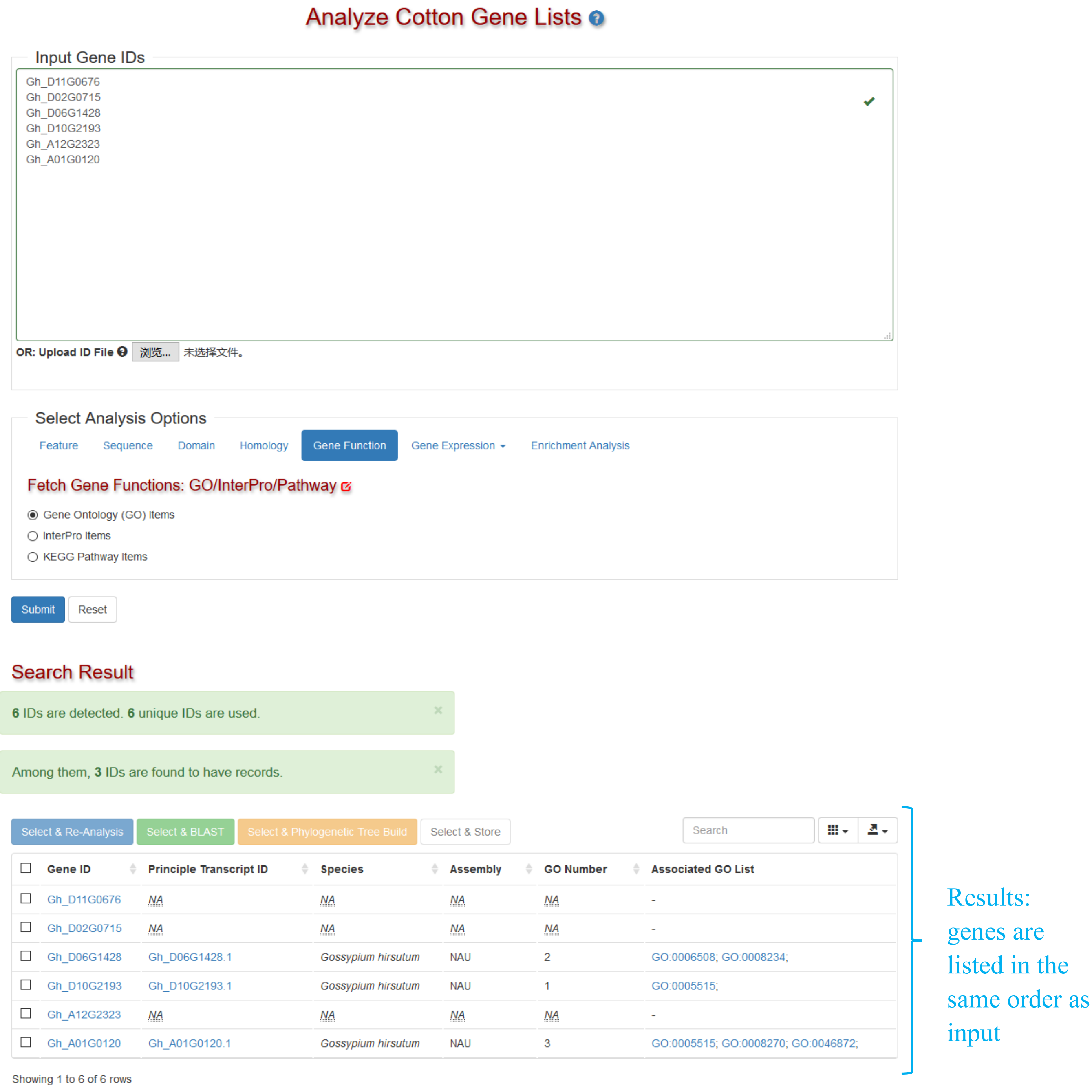

Figure S6-2. Snapshots of the analysis page: fetch function information. This snapshot displayed fetching associated GO terms in six genes in *G. hirsutum* (NAU assembly). Genes are: Gh_D11G0676, Gh_D02G0715, Gh_D06G1428, Gh_D10G2193, Gh_A12G2323 and Gh_A01G0120. Note that even for genes with no GO terms, they are still listed following the original order. Therefore, it is convenient to fetch different information type separately and then connect them together.

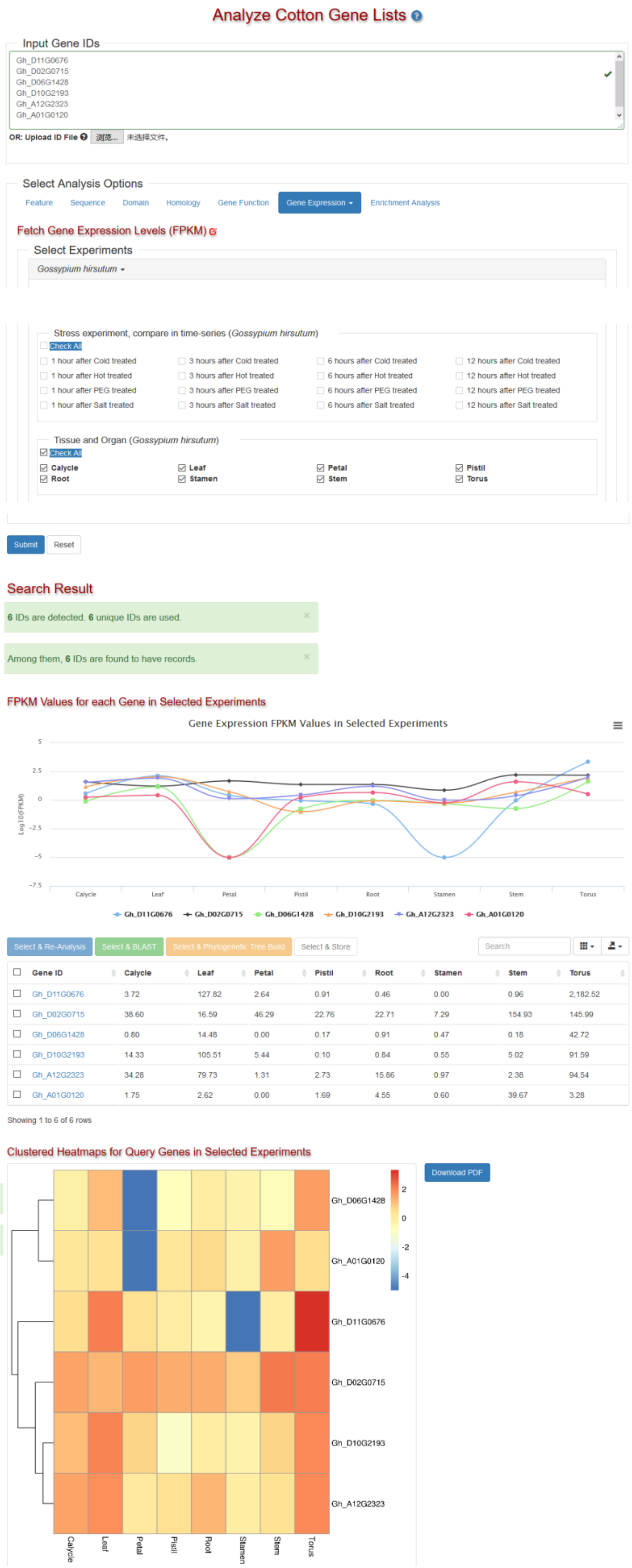

Select experiments (showing partial option only)

List expression level (FPKM) for each gene in selected experiments

Cluster input genes based on their expression atlas in selected experiments

Figure S6-3. Snapshots of the analysis page: fetch expression level information. This snapshot displayed fetching expression levels (FPKM) in six genes of *G. hirsutum* (NAU assembly) in eight organs. Genes are: Gh_D11G0676, Gh_D02G0715, Gh_D06G1428, Gh_D10G2193, Gh_A12G2323 and Gh_A01G0120. In addition to the result table, CottonFGD will generate a heatmap clustered by the expression patterns for these query genes. The cluster is done by the pheatmap library (https://cran.r-project.org/web/packages/pheatmap/index.html).

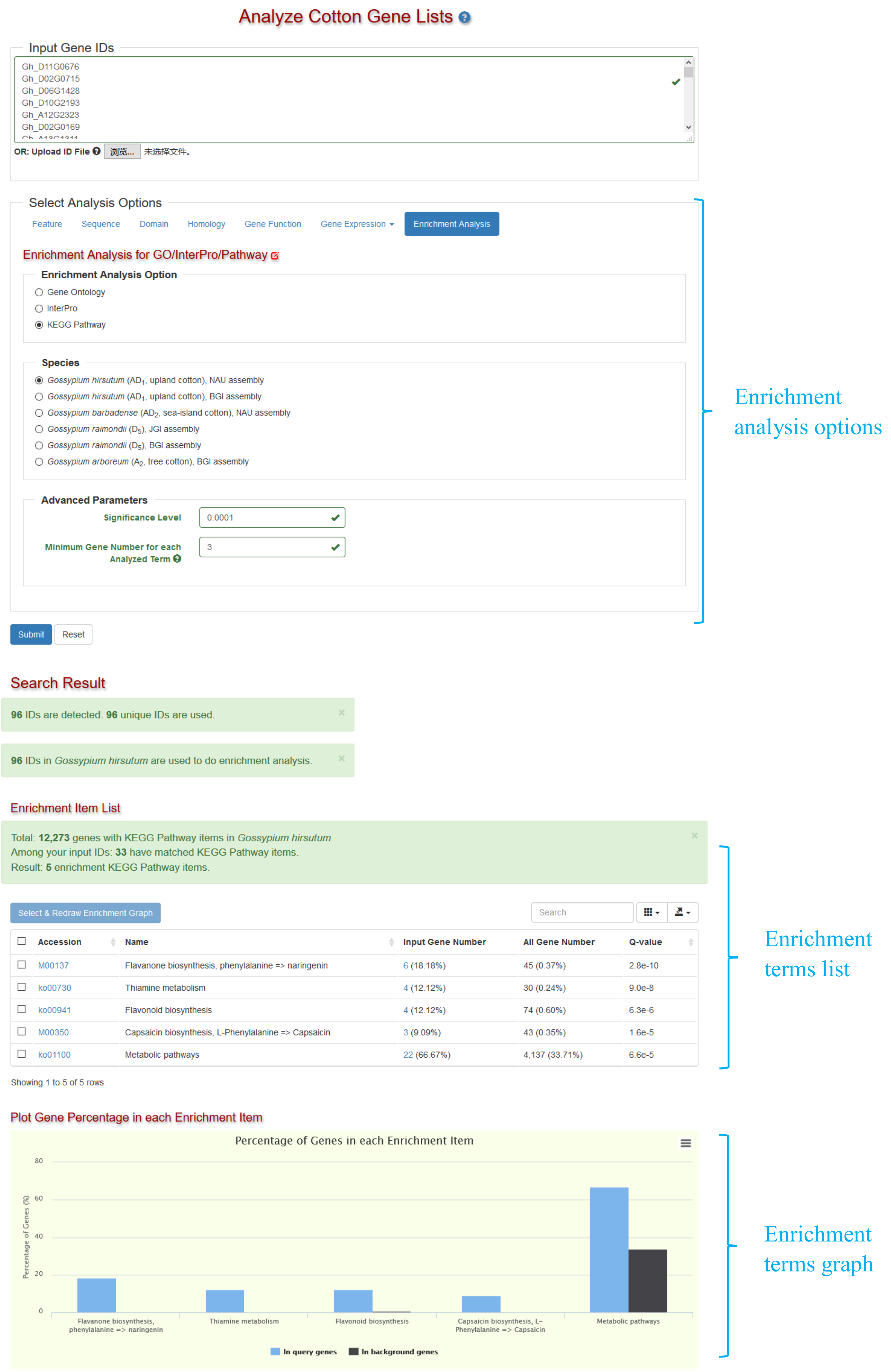
Analyze Cotton Gene Lists
Input Gene IDs
Gh_D11G0676
Gh_D02G0715
Gh_D06G1428
Gh_D10G2193
Gh_A12G2323
Gh_D02G0169
OR: Upload ID File 浏览... 未选择文件。
Select Analysis Options
Feature Sequence Domain Homology Gene Function Gene Expression Enrichment Analysis
Enrichment Analysis for GO/InterPro/Pathway
Enrichment Analysis Option
Gene Ontology
InterPro
KEGG Pathway
Species
Gossypium hirsutum (AD1, upland cotton), NAU assembly
Gossypium hirsutum (AD1, upland cotton), BGI assembly
Gossypium barbadense (AD2, sea-island cotton), NAU assembly
Gossypium raimondii (D5), JGI assembly
Gossypium raimondii (D5), BGI assembly
Gossypium arboreum (A2, tree cotton), BGI assembly
Advanced Parameters
Significance Level 0.0001
Minimum Gene Number for each Analyzed Term 3
Submit Reset
Enrichment analysis options
Search Result
96 IDs are detected. 96 unique IDs are used.
96 IDs in Gossypium hirsutum are used to do enrichment analysis.
Enrichment Item List
Total: 12,273 genes with KEGG Pathway items in Gossypium hirsutum
Among your input IDs: 33 have matched KEGG Pathway items.
Result: 5 enrichment KEGG Pathway items.
Select & Redraw Enrichment Graph
Search
Accession | Name | Input Gene Number | All Gene Number | Q-value
M00137 | Flavanone biosynthesis, phenylalanine => naringenin | 6 (18.18%) | 45 (0.37%) | 2.8e-10
ko00730 | Thiamine metabolism | 4 (12.12%) | 30 (0.24%) | 9.0e-8
ko00941 | Flavonoid biosynthesis | 4 (12.12%) | 74 (0.60%) | 6.3e-6
M00350 | Capsaicin biosynthesis, L-Phenylalanine => Capsaicin | 3 (9.09%) | 43 (0.35%) | 1.6e-5
ko01100 | Metabolic pathways | 22 (66.67%) | 4,137 (33.71%) | 6.6e-5
Showing 1 to 5 of 5 rows
Enrichment terms list
Plot Gene Percentage in each Enrichment Item
Percentage of Genes in each Enrichment Item
Percentage of Genes (%)
0
20
40
60
80
Flavanone biosynthesis, phenylalanine => naringenin
Thiamine metabolism
Flavonoid biosynthesis
Capsaicin biosynthesis, L-Phenylalanine => Capsaicin
Metabolic pathways
In query genes
In background genes
Enrichment terms graph

Figure S6-4. Snapshots of the analysis page: enrichment analysis. This snapshot displayed doing KEGG pathway enrichment analysis in 96 genes of *G. hirsutum* (NAU assembly) using $P < 0.0001$ as thresholds.

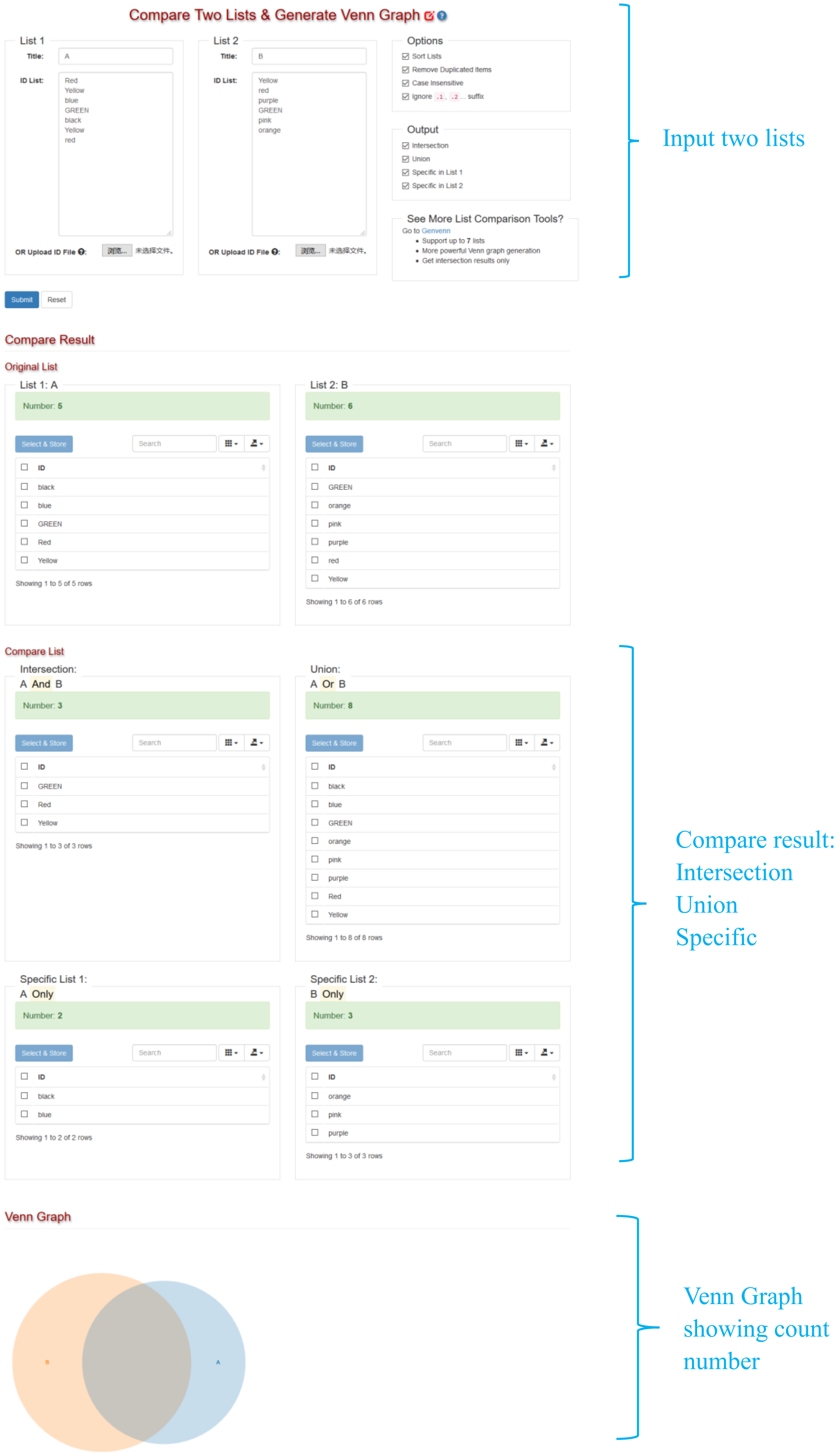
Compare Two Lists & Generate Venn Graph
List 1
Title: A
ID List: Red Yellow blue GREEN black Yellow red
OR Upload ID File
List 2
Title: B
ID List: Yellow red purple GREEN pink orange
OR Upload ID File
Options
Sort Lists
Remove Duplicated Items
Case Insensitive
Ignore .1, .2 ... suffix
Output
Intersection
Union
Specific in List 1
Specific in List 2
See More List Comparison Tools?
Go to Genvenn
Support up to 7 lists
More powerful Venn graph generation
Get intersection results only
Submit
Reset
Input two lists
Compare Result
Original List
List 1: A
Number: 5
ID
black
blue
GREEN
Red
Yellow
Showing 1 to 5 of 5 rows
List 2: B
Number: 6
ID
GREEN
orange
pink
purple
red
Yellow
Showing 1 to 6 of 6 rows
Compare List
Intersection:
A And B
Number: 3
GREEN
Red
Yellow
Showing 1 to 3 of 3 rows
Union:
A Or B
Number: 8
black
blue
GREEN
orange
pink
purple
Red
Yellow
Showing 1 to 8 of 8 rows
Specific List 1:
A Only
Number: 2
black
blue
Showing 1 to 2 of 2 rows
Specific List 2:
B Only
Number: 3
orange
pink
purple
Showing 1 to 3 of 3 rows
Compare result:
Intersection
Union
Specific
Venn Graph
Venn Graph
showing count
number

Figure S6-5. Snapshots of the list compare page. Note this page only conduct comparisons based on inputted IDs. Therefore, it could be applied to any ID lists instead of restricting on cotton gene IDs.

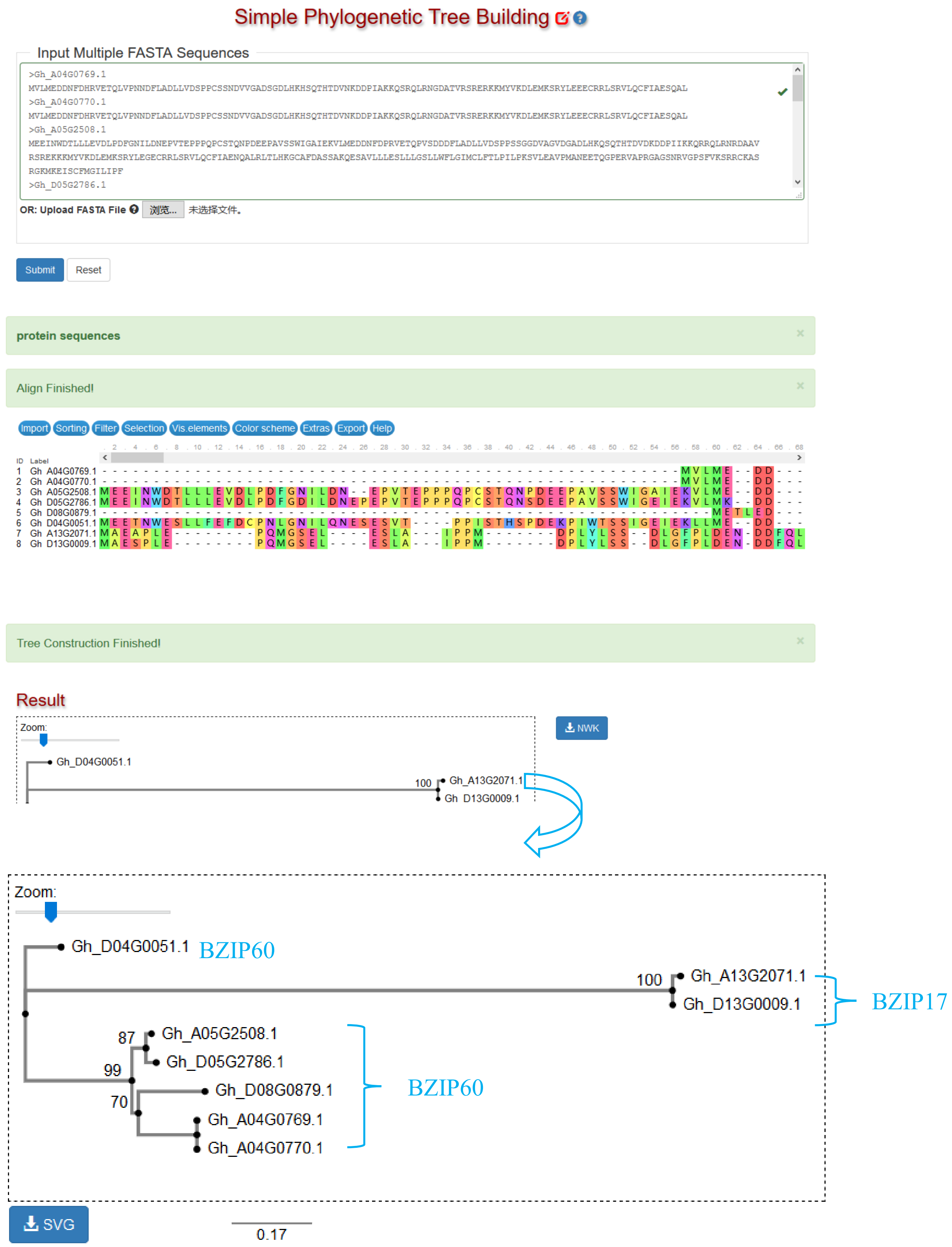

Figure S6-6. Snapshots of the phylogenetic tree build page. This snapshot

displayed a phylogenetic tree built by six *BZIP60* (Gh_A04G0770, Gh_A04G0769, Gh_D04G0051, Gh_A05G2508, Gh_D05G2786, Gh_D08G0879) and two *BZIP17* (Gh_A13G2071, Gh_D13G0009) genes in *G. hirsutum*.
